# Transcriptional signature of CD56^bright^ NK cells predicts favourable prognosis in bladder cancer

**DOI:** 10.3389/fimmu.2024.1474652

**Published:** 2025-01-14

**Authors:** Md Abdullah Al Kamran Khan, Alexander James Sedgwick, Yuhan Sun, Julian P. Vivian, Alexandra J. Corbett, Riccardo Dolcetti, Theo Mantamadiotis, Stefano Mangiola, Alexander David Barrow

**Affiliations:** ^1^ Department of Microbiology and Immunology, The University of Melbourne at The Peter Doherty Institute for Infection and Immunity, Melbourne, VIC, Australia; ^2^ St. Vincent’s Institute of Medical Research, Melbourne, VIC, Australia; ^3^ Department of Medicine, The University of Melbourne, Melbourne, VIC, Australia; ^4^ Australian Catholic University, Melbourne, VIC, Australia; ^5^ Peter MacCallum Cancer Centre, Melbourne, VIC, Australia; ^6^ Sir Peter MacCallum Department of Oncology, The University of Melbourne, Melbourne, VIC, Australia; ^7^ Department of Surgery, Royal Melbourne Hospital, The University of Melbourne, Melbourne, VIC, Australia; ^8^ South Australian immunoGENomics Cancer Institute, The University of Adelaide, Adelaide, SA, Australia; ^9^ Division of Bioinformatics, Walter and Eliza Hall Institute of Medical Research, Parkville, VIC, Australia; ^10^ Department of Medical Biology, University of Melbourne, Parkville, VIC, Australia

**Keywords:** bladder cancer, prognosis, CD56^bright^ NK, NK cells, TCGA, anti-tumour immunity, transcriptional signature

## Abstract

Human natural killer (NK) cells can be sub-divided into two functional subsets but the clinical significance of these CD56^bright^ and CD56^dim^ NK cells in anti-tumour immunity remains largely unexplored. We determined the relative abundances of gene signatures for CD56^bright^ and CD56^dim^ NK cells along with 3 stromal and 18 other immune cell types in the patient tumour transcriptomes from the cancer genome atlas bladder cancer dataset (TCGA-BLCA). Using this computational approach, CD56^bright^ NK cells were predicted to be the more abundant tumour-infiltrating NK subset which was also associated with improved patient prognosis. A similar favorable survival trend was projected using gene signatures for mature myeloid dendritic cells (mDC) and CD8^+^ effector memory T cells (T_EM_) and unveiled a potential CD56^bright^ NK-mDC-CD8^+^T cell crosstalk in the BLCA tumour microenvironment. Expression of transcripts encoding the activating NK cell receptors, NKG2D, NKp44, CD2, and CD160, showed positive survival trends in combination with CD56^bright^ NK cell infiltration. Transcription factors including HOBIT, IRF3, and STAT2 were also correlated with CD56^bright^ NK cell abundance. Additionally, a HOBIT-dependent tissue-residency program correlated with the CD56^bright^ NK and CD8^+^ T_EM_ cell signatures was found to be associated with favourable BLCA patient survival. Overall, our study highlights the significance of CD56^bright^ NK cells in BLCA patient prognosis. Our findings facilitate a better understanding of the NK cell anti-tumour responses that may ultimately lead to the development of promising NK and T cell-based therapies for BLCA.

## Introduction

Bladder cancer (BLCA) is one of the most prevalent cancer types globally, causing approximately 210,000 deaths annually (1). BLCA is classified into two broad clinical categories- non-muscle-invasive bladder cancers and muscle-invasive bladder cancers (MIBCs), where the former is confined to the mucosa or submucosal connective tissue, and the latter also spreads into nearby smooth muscles. Moreover, MIBCs are the most frequent and lethal forms of BLCA (2). Transurethral resection of the bladder tumour in combination with intravesical administration of Bacillus Calmette–Guérin (BCG) can significantly reduce tumour progression, however, frequent recurrence of the tumour even after therapy has been reported (3). BCG vaccination induces the cytotoxic killing of bladder cancer cells by various immune cells including Natural Killer (NK) cells, CD8^+^ T cells, macrophages, and granulocytes (4), yet functional insights into the underlying responses are still elusive. Recent clinical trials using immune-checkpoint blockade (ICB) in BLCA showed promising outcomes, although the clinical responses were limited only to a fraction of MIBC patients (3) due to acquired resistance to ICB (5). Therefore, a better understanding of the immune surveillance mechanisms in BLCA could potentially inform the development of more effective immunotherapies (6).

NK cells are innate lymphoid cells that possess numerous surface activating and inhibitory receptors. The balance of signaling from these opposing receptors controls NK cell activation (7). Conventional NK cells can be subdivided into two major functional subsets, namely CD56^bright^ NK and CD56^dim^ NK cells. The CD56^bright^ NK cell subset is considered as a robust cytokine-producer yet weakly cytotoxic compared to CD56^dim^ NK cells which produce less cytokines but potent cytotoxicity (8). NK cell functions are regulated by the balance of signalling from inhibitory receptors that are specific for MHC class I (MHC-I) and a wide array of activating receptors that can sense ligands upregulated on the surface of tumour cells (9, 10). Thus, NK cells can efficiently target and destroy tumour cells expressing ligands for activating receptors that have also downregulated the expression of surface MHC-I to escape surveillance from cytotoxic CD8^+^ T lymphocytes (11). NK cells also release pro-inflammatory cytokines, such as IFN-γ and TNF, which can mediate anti-proliferative, anti-angiogenic and pro-apoptotic effects on tumour cells (12), as well as regulate the anti-tumour functions of other immune cells in the tumour-microenvironment (TME) (13). Thus, NK cells are thought to contribute to tumour surveillance *in vivo*, but the relative contributions of the CD56^bright^ and CD56^dim^ NK cell subsets to anti-tumour immunity in human cancer patients remains unclear.

Despite being a minor population amongst the tumour-infiltrating lymphocytes (TILs), the presence and prognostic relevance of NK cells has been observed in many solid cancers, such as head and neck squamous cell carcinoma, non-small cell lung carcinoma, metastatic melanoma, colorectal carcinoma, gastric cancer, and oesophageal cancers (14, 15). Furthermore, studies have also reported the potential implications of the tissue-resident NK subset in lung cancer (16–19), pancreatic cancer (20), and ovarian cancer (21). Also, an abundance of CD56^bright^ NK cell subset was observed in non-small cell lung cancer which had increased expression of CD69, a lymphocyte activation and tissue-residency marker (22). Interestingly, Mukherjee and colleagues previously reported the association of CD56^bright^ NK cells with favourable BLCA patient survival using flow cytometric estimation of TILs (23). A similar prognostic effect was reported for a transcriptional signature of IL-2 activated NK cells in BLCA (24). Nonetheless, the functional role and clinical significance of the specific NK subsets in BLCA are yet to be elucidated. Here, we report the relative abundance of tumour-infiltrating CD56^bright^ and CD56^dim^ NK cells in BLCA using subset-specific transcriptional signatures and correlate these gene signatures with clinical outcomes in BLCA. Collectively, we describe a protective CD56^bright^ NK-mDC-CD8^+^T cell crosstalk signature in the BLCA tumour microenvironment.

## Materials and methods

### Generation of transcriptional signatures of CD56^bright^ and CD56^dim^ NK cells

We obtained the RNA-seq datasets for CD56^bright^ NK cells and CD56^dim^ NK cells along with 3 stromal cell types (i.e., epithelial, endothelial, and fibroblast cells), and 18 other immune cell types (i.e., mast cells, memory B cells, naïve B cells, eosinophil, monocyte, neutrophil, γδ-T cells, immature myeloid dendritic cells, mature myeloid dendritic cells, M1-macrophages, M2-macrophages, naïve CD8^+^ T-cells, helper T-cells, regulatory T-cells, central CD4^+^ memory T-cells, effector CD4^+^ memory T-cells, central CD8^+^ memory T-cells, and effector CD8^+^ memory T-cells) from the Human Bulk Cell-type Catalogue (HBCC) (25) database. To adjust the gene-transcript abundance for the intra and inter-dataset variability, we used a Bayesian multilevel noise modelling approach, Cellsig (25). Cellsig used the cellular differentiation hierarchy to compute the missing transcript-abundances for every node in the hierarchy (**Supplementary Figure S1**).

We used the adjusted cell type transcriptomes from Cellsig to generate the transcriptional signature matrix (**Supplementary Table S1**) using CIBERSORTx (26); with G.min = 25, G.max = 30, and q.value = 0.01, while the rest of the parameters were kept default. To assess the specificity of the transcriptional signature, we performed principal component analysis (PCA) on the HBCC dataset with the identified cell type markers.

### Estimating the abundances of tumour-infiltrating cell types and association with BLCA patient prognosis

We obtained the bulk RNA-seq datasets and the matched clinical information of BLCA patients from the cancer genome atlas (TCGA) through the GDC Data Portal (27). We removed any duplicated sample-transcript pairs within the retrieved data, then scale-normalised the transcript-abundances for the sequencing depths by trimmed mean of M values (TMM) method (28). We selected only the transcriptomes of primary tumour (n = 408) and adjacent normal tissue samples (n = 19) for the analysis. To estimate the relative abundances of the tumour-infiltrating immune cells, we applied the cellular deconvolution method on the patient transcriptomes with the CIBERSORT deconvolution algorithm (29) implemented in the tidybulk R-package (30). We used our generated cell type signature matrix as the deconvolution reference, while the other parameters were kept default.

To correlate the cell type signature abundances with the patient outcomes, we carried out survival analysis with Kaplan–Meier (KM) estimates with survminer (31) package, where the patients were split into groups (i.e., high, and low) based on the median abundance of a cell type abundance. The differences between the survivals of the groups were compared with log-rank (Mantel–Cox) test (32). P-values of the KM curves were adjusted following the Benjamini-Hochberg’s (BH) method (**Supplementary Table S2**).

### Differential gene expression analysis

We conducted differential gene expression analysis to contrast between different patient groups with the DESeq2 (33) package implemented in tidybulk (30). Additionally, the p-values of the contrasts were multiple-test corrected with Benjamini-Hochberg’s (BH) false discovery rate (FDR) method (34). After the contrast, we only selected the genes with |log_2_FoldChange| ≥ 1 and adjusted p-value < 0.05 as significantly differentially expressed (DE).

### Functional enrichment analysis

Pathways associated with a target gene set were identified using with functional overrepresentation analysis (ORA) (35) by clusterProfiler (36) package. We performed the analysis against various pathway modules, such as gene ontology biological process (GOBP) (37), KEGG (38), Reactome (39), Wikipathways (40), and BioPlanet pathway database (41). Enriched pathways with p-values < 0.05 were considered significant.

### Analysis of single-cell RNAseq dataset

We also obtained an independent single-cell RNAseq dataset (GSE135337) (42) from the Gene Expression Omnibus (GEO) database (43). This dataset recorded the single cell transcriptome from 7 bladder cancer patients with varying tumour stages and grades. The patients were newly diagnosed and did not receive any prior treatments. We analysed this dataset with the Seurat (44) package. While preprocessing, we kept only the high-quality cells that satisfy the criteria of nFeature_RNA > 200, nFeature_RNA < 5000, and percentage of mitochondrial transcripts < 10. Also, we removed the ribosomal transcripts from the count information. All samples were pre-processed and then normalised by the SCTransform method with v2 regularization separately before we merged them together with SCTransform. The top 15000 features per sample were used to identify the integration anchor set for the merging. After the integration of the samples, principal component analysis (PCA) was performed up to the first 15 dimensions. To cluster the cells, first the neighbour cells were searched through the shared nearest-neighbor (SNN) graph approach with default parameters. Then the cells were clustered through the Leiden algorithm in igraph method with a clustering resolution of either 0.2 or 0.6. After the clustering, we identified the cluster-specific markers by the Wilcoxon Rank Sum test with a log-FoldChange threshold of 0.25 (**Supplementary Table S3**). In order to identify the characteristics of the clusters, SingleR (45) package was used where the cell type marker references were taken from the EncodeBlueprint database. To assess the presence of CD56^bright^ and CD56^dim^ NK signatures, the clusters were screened using the AddModuleScore function. To obtain the ligand-receptor interactions and estimate the communications between the clusters, cellchat (46) package was utilised.

### Statistical analysis

Statistical significance of the comparison between the means between two groups in the boxplots were achieved through the non-parametric Wilcoxon signed-rank test (47), while comparison of means between multiple groups was performed by non-parametric ANOVA (Kruskal–Wallis) test (48).

## Results

### CD56^bright^ NK cells are associated with favourable prognosis in bladder cancer patients

**Figure 1:**
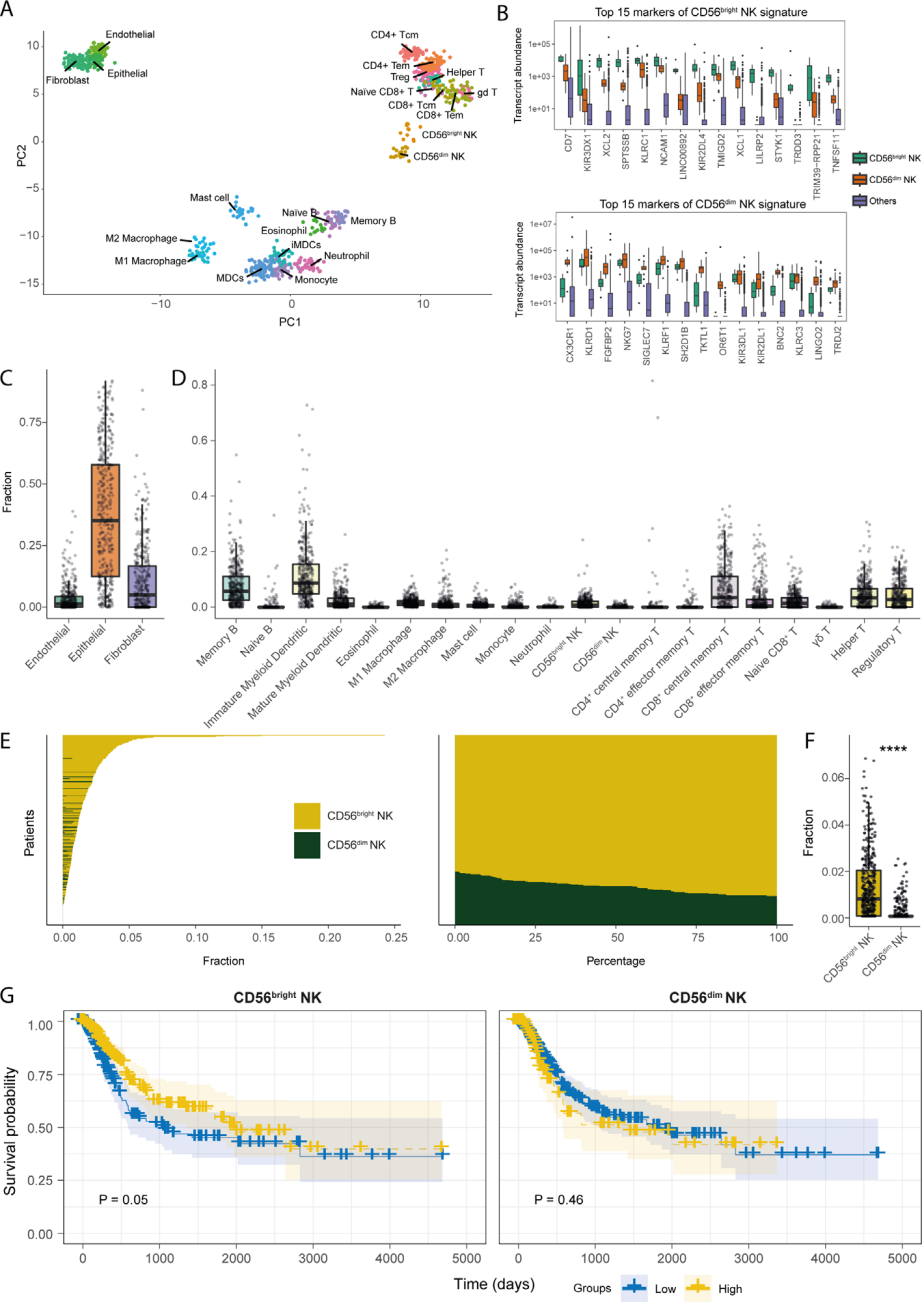
Transcriptional signatures of CD56^bright^ NK cells are associated with favourable prognosis in BLCA patients. **(A)** PCA plot for the samples of the cell types included in the transcriptional signature reference; **(B)** Boxplots representing the expression comparisons of top 15 markers of CD56^bright^ and CD56^dim^ NK cells. Estimated fractions of the **(C)** stromal, and **(D)** immune cell types in BLCA primary tumour tissue samples; **(E)** Patient-wise NK subset fractions and their relative amounts of total NK infiltration; **(F)** Bar plot comparing the estimated NK subset fractions; **(G)** KM-curves for the NK subsets; high abundance of CD56^bright^ NK cell correlates with better BLCA patient survival as opposed to CD56^dim^ NK cell. (****p-value < 0.0001 for Wilcoxon signed ranked test).

NK cells are thought to play a significant role in cancer immune surveillance through direct cytotoxicity of tumour cells and immunomodulation (49, 50). We hypothesized that subset-specific functionalities of NK cells may be present in the BLCA TME that could regulate anti-tumour immunity. To address this, we first sought out the core transcriptional markers of CD56^bright^ and CD56^dim^ NK cells that segregate these phenotypic subsets from each other, and from 21 other stromal and immune cell types, including T lymphocyte subsets (**Figures 1A, B**). In the PCA analysis with the generated transcriptional signatures, a clear contrast between stromal and immune cell subsets was observed (**Figure 1A**). Within immune cells, naïve and memory B cells clustered more closely with the myeloid cell subsets, whereas the NK cell subsets clustered more closely with T lymphocyte subsets. Importantly, the NK cell subsets formed distinct clusters separated from each other and from all other immune cell subsets, including the CD8^+^ T cell subsets (**Figure 1A**). Moreover, the classical gene markers for CD56^dim^ and CD56^bright^ NK cells showed preferential subset-specific expression patterns, respectively (**Figure 1B**). These results show that we have identified gene signatures specific for CD56^dim^ and CD56^bright^ NK cell subsets that can be distinguished from each other and from other immune and stromal cell-types.

We next estimated the relative abundances of the tumour-infiltrating NK subsets in the TCGA-BLCA patient cohort by cellular deconvolution. Bladder cancers are primarily of epithelial origin (51). Indeed, we found epithelial cells to be the most abundant stromal cells in the BLCA TME followed by fibroblasts (**Figure 1C**). Amongst, immune cell types, signatures of immature dendritic cells, memory B cells, and CD8^+^ central memory T cells were the most abundant in BLCA (**Figure 1D**). Interestingly, we observed the CD56^bright^ NK cell signature to be the major infiltrating NK subset in BLCA patients (**Figures 1E, F**). Furthermore, patients from different histopathological grades and stages also harboured similar NK subset infiltration profiles (**Supplementary Figures S2A–E**). Intriguingly, patients with more advanced tumour-stages (e.g., higher grades, and higher pathologic stages) showed significantly higher abundance of infiltrating CD56^bright^ NK cells, but not CD56^dim^ NK cells. Additionally, a similar enrichment of CD56^bright^ NK signatures was present in the BLCA tumour-adjacent normal tissues (**Supplementary Figure S3A**). These findings indicate that CD56^bright^ NK cells may preferentially infiltrate BLCA tumours compared to CD56^dim^ NK cells.

We also analysed an independent single-cell RNAseq dataset which includes transcriptomes of 7 muscle-invasive bladder cancer patients. We found that signatures for epithelial cells followed by fibroblasts were most abundant within the tumour, consistent with our findings from the deconvolution of TCGA-BLCA dataset (**Supplementary Figure S3B**). We then selected only the CD45^+^ cells and performed clustering to identify the tumour-infiltrating immune cell populations. From this, we obtained 8 clusters representing different immune cell populations (**Supplementary Figure S3C**). Upon annotating the phenotypic characteristics of the clusters, cluster 6 bore the highest resemblance to CD8^+^ T and NK cells (**Supplementary Figure S3D**). Additionally, amongst all the clusters, cluster 6 contained the phenotypic signatures of both NK subsets (**Supplementary Figure S3E**). We also detected that the presence of NK subset signatures in cluster 6 was significantly enriched compared to the average of background signatures for other immune cells used in the deconvolution of the TCGA bulk transcriptome data (**Supplementary Figures S3F, G**).

We next assessed the potential implications of the infiltrating NK subsets for BLCA patient prognosis, which revealed an association between a higher abundance of CD56^bright^ NK cells, but not CD56^dim^ NK cells, with improved BLCA patient survival (**Figure 1G**). Also, CD56^bright^ NK cells were predicting a trend, although not significant, towards the favourable patient survival even after associating other important clinical variables such as high mutational burden and CD8^+^ T cell abundance (**Supplementary Figure S4**). Additionally, the favourable prognostic implications from CD56^bright^ NK cell infiltration can greatly differ between patients with varying clinicopathologic tumour status. Interestingly, this positive prognostic association of CD56^bright^ NK cells was more prominent in patients with moderately developed non-metastatic (e.g., pathologic T2, T3, N0, M0, stage II, and stage III) tumours; as opposed to the patients with more advanced and metastatic, suggesting a potential anti-tumour role of CD56^bright^ NK cells in the BLCA TME (**Supplementary Figures S5A–D**).

### Expression of NK receptors is associated with improved BLCA prognosis

**Figure 2:**
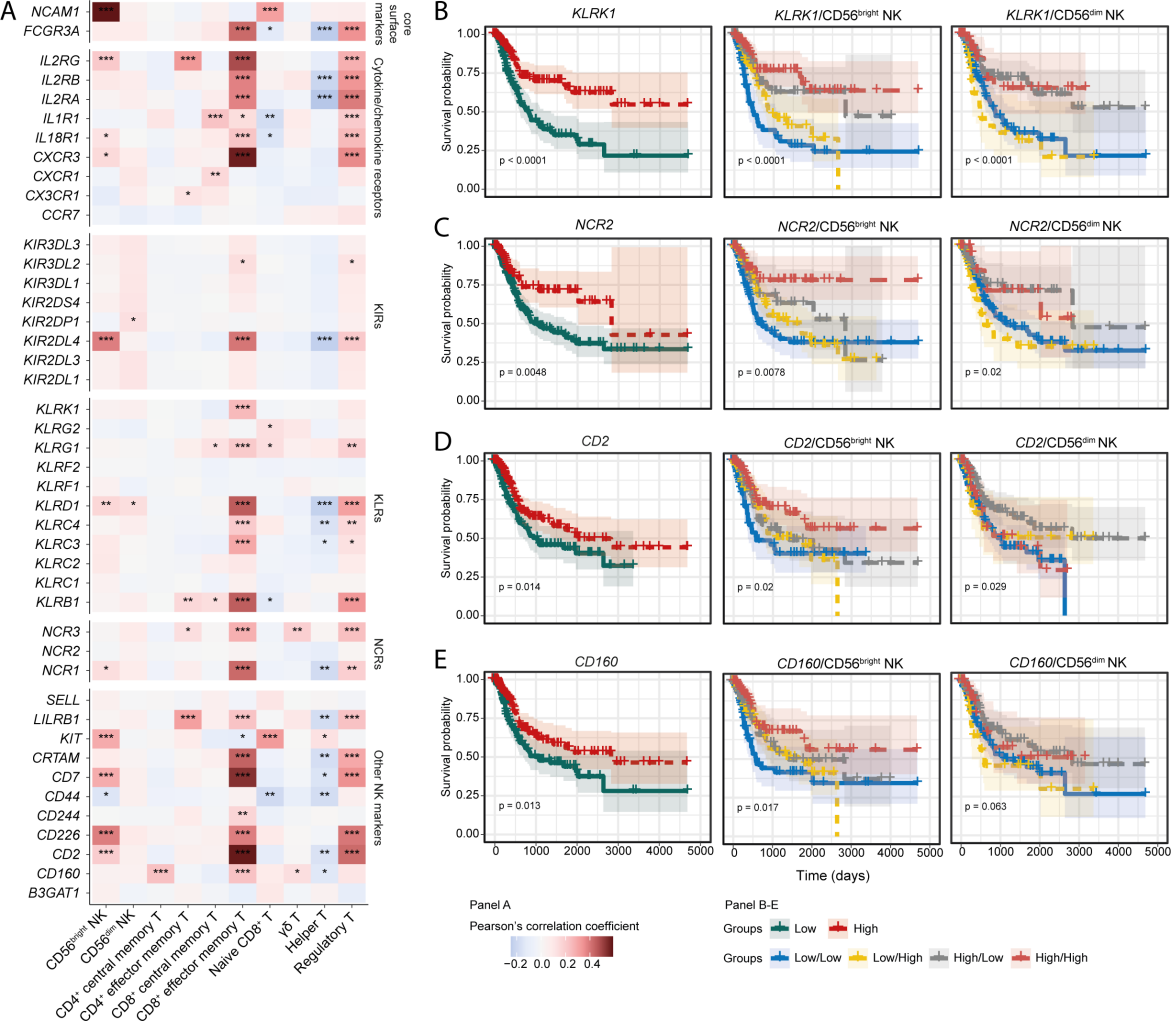
Expression of NK-associated signaling receptor are associated with NK cell subset signatures. **(A)** Correlation heatmaps for showing the correlation between different NK receptors and NK/T cell subsets; KM curves to highlight the prognostic significance of **(B)** *KLRK1* (NKG2D), **(C)** *NCR2* (NKp44), **(D)** *CD2*, and **(E)** *CD160* receptor genes. (***p-value < 0.001, **p-value < 0.01, *p-value < 0.05 for Pearson’s correlation coefficient scores).

Activation of NK cell functionality is dictated by the signaling through activating and inhibitory surface receptors. However, which specific NK family receptor(s) drive NK cell anti-tumour responses in human cancer patients is understudied. Since the signature of CD56^bright^ NK cells is correlated with favourable BLCA patient survival, we next determined whether expression of NK family receptors is similarly important. Tumour-specific expression of the NK receptors *NCAM1*, *IL2RG*, *IL18R1*, *CXCR3*, *KIR2DL4*, *KLRD1*, *NCR1*, *KIT*, *CD7*, *CD2*, and *CD226* showed a positive correlation with the infiltrating CD56^bright^ NK subset, but not with CD56^dim^ NK cells (**Figure 2A**). Intriguingly, many of these receptors were also positively correlated with the signature of CD8^+^ effector memory T cells (CD8^+^ T_EM_) (**Figure 2A**). The expression of *KLRK1* (NKG2D), *NCR2* (NKp44), *CD2*, and *CD160* receptor genes were associated with favourable prognosis (**Figures 2B–E**). Moreover, patients harbouring higher expression of these receptors alongside higher CD56^bright^ NK abundance showed trends towards better survival compared to others (**Figures 2B–E**). However, involvement of these receptors with the CD56^dim^ NK subset was not conclusive (**Figures 2B–E**). Multivariate analysis also highlighted the prognostic associations of these receptors with the signatures of CD56^bright^ NK cell (**Supplementary Figure S6A**). Similar survival trends for *KLRK1* (NKG2D), *CD2*, and *CD160* expression were also predicted in combination with the CD8^+^ T_EM_ cells (**Supplementary Figure S6B**).

Next, we asked whether the ligands for NK family receptors are also positively associated with the infiltrating immune cell signatures. Correlation analysis indicated positive associations of CD160 ligands such as *TNFRSF14* and *HLA-C*, and NKG2D (*KLRK1*) ligands *MICA* and *MICB* with the tumour-infiltrating CD56^bright^ NK and CD8^+^ T_EM_ cells (**Supplementary Figure S6C**). Previously, NKG2D (*KLRK1*) ligands MICA, and MICB were shown to have associations with beneficial BLCA patient prognosis (24). Expression of the CD160 ligands trended towards favorable prognosis, whereas the ligands of NKp44 (*NCR2*) were associated with improved survival (**Supplementary Figure S6D**). Compared to the BLCA tumour-adjacent normal tissues, tumour expression of transcripts encoding CD2, KLRK1, and NCR2 were higher (**Supplementary Figure S6E**). Also, in the analysed BLCA scRNA-seq data, most of the NK cell markers were expressed by the cells of cluster 6 to some extent; except for *CD44*, expression which was also observed in other clusters (**Supplementary Figure S6F**). These results highlight that signaling through NKG2D (*KLRK1*), NKp44 (*NCR2*), and CD160 receptors may be critical in the downstream activation of tumour infiltrating CD56^bright^ NK and/or CD8^+^ T_EM_ cells, which may contribute to a more favourable BLCA prognosis.

### Cooperation between NK cells, myeloid dendritic cells, and CD8^+^ T cells may be crucial in bladder cancer prognosis

The anti-tumour functions of NK cells may be directed by other cells in the TME. Conversely, NK cells can also influence the recruitment and functional activation of other tumour-infiltrating leukocytes (52). We next sought to determine the immune cell types that may be critical to BLCA patient prognosis, and their potential association with the CD56^bright^ and CD56^dim^ NK subsets.

**Figure 3:**
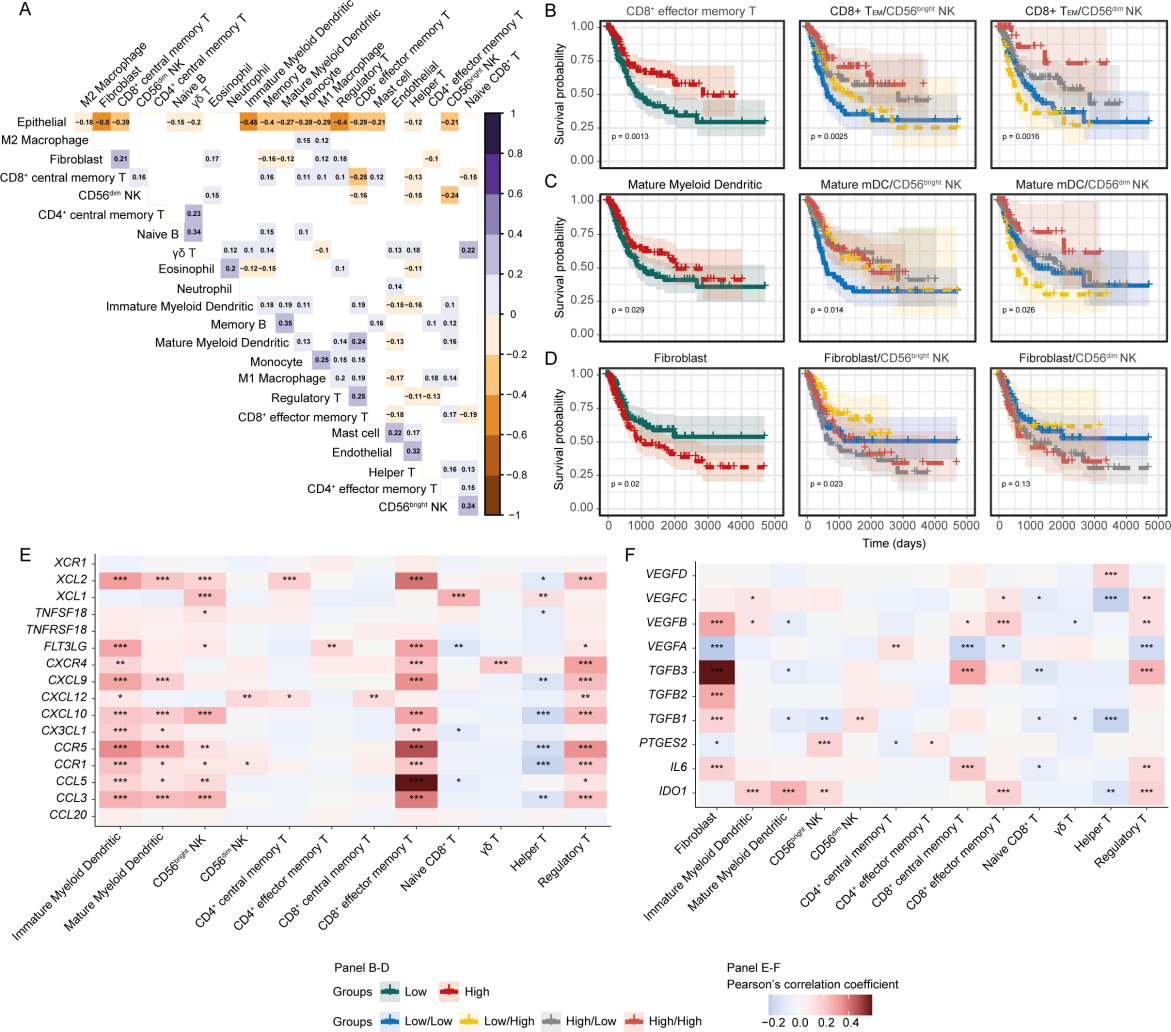
Crosstalk between CD56^bright^ NK and other cells might shape up the immune outcomes. **(A)** Heatmap showing the pair-wise correlation between the abundances of cell subsets; **(B)** Prediction of survival associations of **(B)** CD8^+^ T_EM_, **(C)** mDCs, and **(D)** Fibroblasts independently and in combination with NK subsets. Correlation heatmap for the functional molecules involved in **(E)** NK-mDC-CD8^+^ T, and **(F)** Fibroblast-immune cell cross-talks. (***p-value < 0.001, **p-value < 0.01, *p-value < 0.05– for Pearson’s correlation coefficient scores).

Pair-wise correlation between the signature abundances of the immune and stromal cell subsets revealed an intriguing inverse correlation between the two NK subsets (**Figure 3A**). Additionally, the signature of CD56^bright^ NK cells was positively associated with immature and mature myeloid dendritic cells (imDCs and mDCs), Memory B, M1 macrophage, Naïve CD8^+^, and CD8^+^ T_EM_ cells. In contrast, CD56^dim^ NK was positively correlated with infiltrating central CD8^+^ memory T cells (CD8^+^ T_CM_), but negatively with CD8^+^ T_EM_ and T helper cells (**Figure 3A**). This finding suggests that the infiltrating CD56^bright^, and CD56^dim^ NK cells are associated with distinct immune signaling networks within the tumour.

We next asked whether the associations between the NK subsets and other cell types might influence patient survival. First, we determined the prognostic association of each cell type separately and observed favourable survival associations for CD8^+^ T_EM_ and mDCs, and poor survival association for fibroblast cells (**Figures 3B–D**). Next, we investigated the survival associations of these cells in combination with the NK subsets and found that BLCA patients with high CD56^bright^ or CD56^dim^ NK with high CD8^+^ T_EM_ and mDCs signatures had improved survival (**Figures 3B, C**). Cancer-associated fibroblasts (CAFs) can potentially dampen the anti-tumour functions of tumour-infiltrating NK cells (53). We observed that patients with high fibroblasts but low CD56^bright^ NK had the worst survival outcome, with this effect not as pronounced for CD56^dim^ NK cells (**Figure 3D**). This highlights that the protective effect that correlates with these immune signatures are tumour environment -dependent. To support this finding, we estimated each of the cell type signatures using gene set enrichment approach independently and mapped it onto the BLCA patient clusters that was generated from the estimated abundances from the deconvolution. Interestingly, we observed that a group of BLCA patients were highly enriched for the CD56^bright^ NK signature as well as for CD8^+^ T_EM_ and mDCs signatures. Also, a collinearity was present between the abundances of these cell signatures in the BLCA patients (**Supplementary Figures S7A–E**). These further supports the putative NK-mDC-CD8^+^ T signalling axis in BLCA tumour microenvironment. We also sought to detect the putative immune crosstalk in the analysed BLCA scRNA-seq data. Despite many potential ligand-receptor interactions between cluster 6 cells with the immune cells from other clusters, communication between the cells was slightly ambiguous due to the limited number of immune cells in the data (**Supplementary Figures S3H–J**).

In the TME, complex interactions between immune cells can occur through a wide-variety of secreted factors (52). Considering our identified NK-mDC-CD8^+^ T signaling axis, we extended our analysis to encompass the effect of genes encoding secreted factors in the BLCA TME. We detected a strong positive correlation between the expression of cytokine-chemokines, and their receptor encoding genes and myeloid dendritic cells, CD56^bright^ NK and CD8^+^ T_EM_ cell phenotypes (**Figure 3E**). Conversely, expression of fibroblast-associated factors, such as *VEGFB*, *VEGFC*, *TGFB1*, *TGFB2*, *TGFB3*, and *IL6* were positively associated with the fibroblast signature, but were negatively correlated with the infiltrating myeloid dendritic cell, NK, and T-cell subset signatures (**Figure 3F**); indicating that cancer-associated fibroblasts might be negatively modulating the anti-tumour immune cell functions (54). Overall, these results highlight the presence of complex anti- and pro-tumorigenic interactions in the BLCA TME that may shape the anti-tumour function of the CD56^bright^ NK-mDC-CD8^+^T cell axis.

### Anti-tumorigenic gene pathways are associated with the CD56^bright^ NK signature

**Figure 4:**
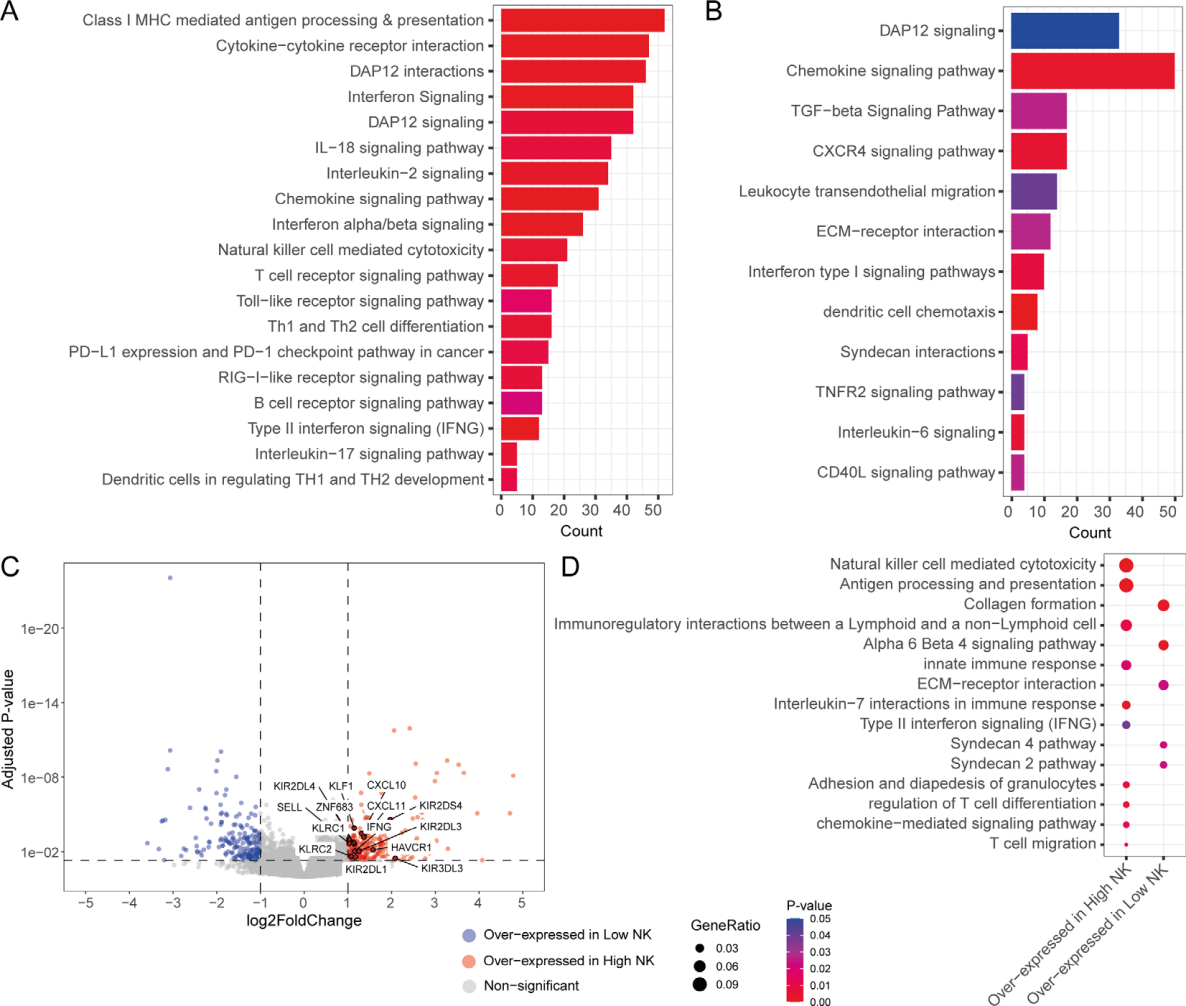
Tumour-infiltrating NK subsets are associated with unique transcriptomic responses. Functional pathways associated with **(A)** CD56^bright^ NK and **(B)** CD56^dim^ NK infiltration signatures; **(C)** Volcano plot showing the differential expressed DE genes from the contrast between high and low total NK group. DE genes with |logFC| >1 and adjusted p-value < 0.05 were considered significant; **(D)** Functional pathways associated with the overexpressed genes in high and low total NK groups.

To obtain insights into the functional mechanisms associated with the BLCA-infiltrating NK subsets, we sought to understand the molecular pathways associated with our CD56^bright^ and CD56^dim^ signatures. We performed functional pathway analysis with genes that are positively correlated with the abundance of the NK cell subsets. Various immune signaling pathways were positively associated with the CD56^bright^ NK signature, including pathways associated with NK cell-mediated responses, interferon signaling, T and B cell maturation and signaling, and cytokine-chemokine signaling (**Figure 4A**). In contrast, the CD56^dim^ NK signature was weakly associated with interferon and other cytokine-chemokine signaling, and dendritic cell recruitment pathways (**Figure 4B**). Surprisingly, several pro-tumour pathways such as TGF-β signaling, ECM remodelling, and IL-6 signaling were associated with CD56^dim^ NK cell abundance, suggesting these pathways suppress the functions of this NK subset.

We also sought out the differences in the functional responses between the patients with high and low/no NK cell infiltration. To achieve this, we split the patients into three groups (e.g., high, medium, and low) based on total NK signature abundance. Then we compared the transcriptomes of the high total NK against low total NK group through a differential gene expression analysis. The high total NK group was found to over-express key NK function associated genes such as *SELL*, *IFNG*, *KLRC2* (NKG2C), *KIR2DS4*, *CXCL10*, *ZNF683*, and *CXCL11* (**Figure 4C**). Remarkably, this patient group also expressed high amounts of NK family receptors such as *KLRC1* (NKG2A), *KIR2DL1*, *KIR2DL3*, *KIR2DL4*, *KIR3DL3* and *HAVCR1* (TIM1) (**Figure 4C**). Pathway analysis with the differentially expressed genes revealed that immune signaling pathways (such as NK cell mediated cytotoxicity, antigen processing and presentation, IL-7 interactions in immune responses, Type II interferon signaling, and chemokine mediated signaling pathways) were mostly associated with the high NK infiltration group in contrast to the low NK infiltration group, which had upregulated expression of pro-tumour pathways such as fibroblast-associated pathways (e.g., collagen production), and ECM remodelling pathways (**Figure 4D**). Many of these pathways were also associated with the cells of NK/CD8^+^ T cell cluster (Cluster 6) of the analysed BLCA scRNA-seq dataset (**Supplementary Figure S8**). These findings indicate that BLCA patients with low overall NK infiltration had more pro-tumorigenic responses as opposed to the patients with high overall NK abundances where more anti-tumorigenic pathways were present.

### Transcription factors involved in NK and T cell function and development might facilitate favourable BLCA patient survival

**Figure 5:**
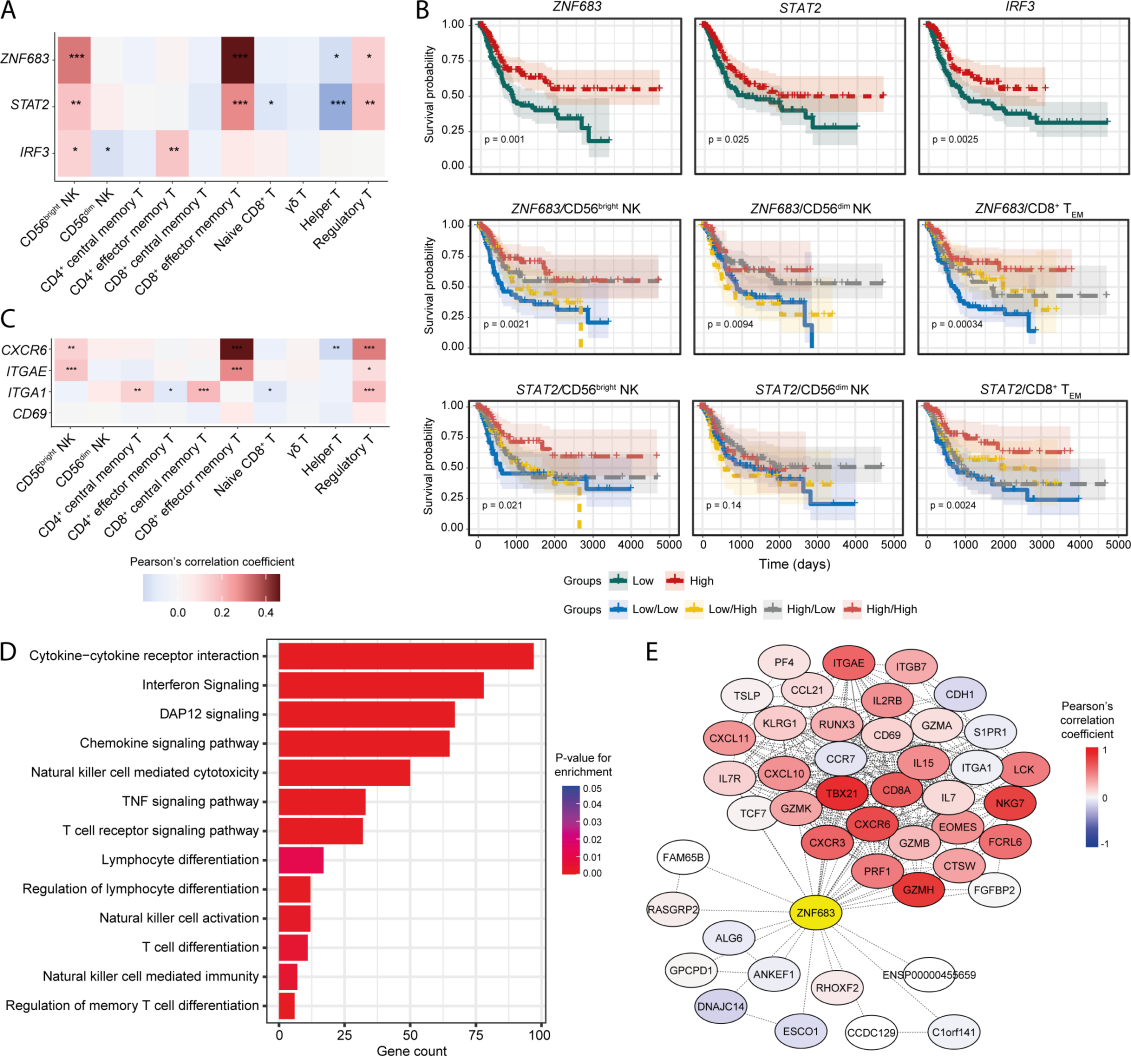
Transcription factors involved in NK/T-cell development and function are associated with infiltrating NK and CD8^+^ T cell signatures. **(A)** Heatmap showing the correlation of *ZNF683* (HOBIT), *STAT2*, and *IRF3* expression with infiltrating NK and T-cell abundances; **(B)** Prognostic associations of these TFs independently and in combination with NK subsets and CD8^+^ T_EM_ cell; **(C)** Correlation of markers of tissue-residency program with abundance of infiltrating NK and T-cell subsets; **(D)** Functional pathways associated with *ZNF683* (HOBIT) expression; **(E)** Correlation of *ZNF683* (HOBIT) expression with HOBIT-associated functional molecules. (***p-value < 0.001, **p-value < 0.01, *p-value < 0.05– for Pearson’s correlation coefficient scores).

Transcription factors (TFs) can often play key roles in modulating the pro-/anti-tumour functions of immune cells (55–57). Therefore, we searched for the transcription factors that are positively correlated with the NK signatures and explored their possible impact on patient survival. Positive associations of several TF-encoding genes such as *ZNF683* (HOBIT), *STAT2*, and *IRF3* with the abundances of tumour-infiltrating CD56^bright^ NK and CD8^+^ T_EM_ cells were observed (**Figure 5A**). Transcript abundance of these TFs was significantly higher in the BLCA tumour tissues compared to the adjacent normal tissue (**Supplementary Figure S9A**). Additionally, these TFs were significantly associated with favourable patient survival, and patients with higher expression of these TFs alongside higher CD56^bright^ NK and CD8^+^ T_EM_ cells, but not CD56^dim^ NK cells, abundances had better survival probabilities (**Figure 5B**, **Supplementary Figure S9B**). Furthermore, multivariate analysis suggests the potential co-implications of *ZNF683* and CD56^bright^ NK cell signature in favourable BLCA patient prognosis (**Supplementary Figure S9C**). These results identify TF signatures that may serve as markers of modulation of anti-tumour functions of tumour-infiltrating NK and T cell subsets in BLCA.

HOBIT has been shown to regulate a program of tissue-residency in CD8^+^ T lymphocytes (58). We hypothesized that expression of HOBIT might also dictate tissue-residency programming in tumour-infiltrating NK cell subsets. Interestingly, both the CD56^bright^ NK and CD8^+^ T_EM_ cell signatures were strongly correlated with the expression of tissue-residency markers such as *ITGAE* (CD103), and *CXCR6* (**Figure 5C**), while *CD69* expression was weakly correlated, and no correlation was observed for *ITGA1* (CD49a) with CD56^bright^ NK and CD8^+^ T_EM_ cells (**Figure 5C**). Overall, these findings suggest the presence of a tissue-resident phenotype of NK and/or T cells in the BLCA TME.

Next, we detected numerous lymphocyte development and function associated pathways that are significantly associated with *ZNF683* (HOBIT), *STAT2*, and *IRF3* expression (**Figure 5D**, **Supplementary Figures S9D, E**), indicating that these TFs may regulate the priming, and their downstream functionalities of tumour-infiltrating NK and CD8^+^ T cells. Also, expression of the NK associated receptors was correlated with the expression of these TFs, indicating the potential influence of these TFs on the functional responses on NK and CD8^+^ T cell subsets (**Supplementary Figure S9F**). Also, expression of *ZNF683* (HOBIT) as well as markers of tissue-residency were detected in a fraction of the cluster 6 population in the BLCA scRNA-seq dataset (**Supplementary Figure S9G**) supporting the presence of a tissue-resident NK or T cell population in the bladder TME.

Furthermore, we detected numerous genes known to be regulated by the HOBIT transcription factor that were co-expressed with *ZNF683* (HOBIT) in the BLCA patients (**Figure 5E**). EOMES regulates the tissue-residency program of NK and CD8^+^ T cells alongside HOBIT (59, 60). We observed a positive correlation between *ZNF683* (HOBIT) and tissue-retention markers such as CD69 and CD103 (*ITGAE*) that are essential for the establishment of tissue-resident phenotypes in NK and T cells (61–63). On the other hand, tissue-egress factors such as S1PR1 and CCR7 (64) were found to be negatively correlated with *ZNF683* (HOBIT) expression. These results suggest that HOBIT may regulate TIL phenotype in the BLCA TME by driving a tissue-residency program in tumour-infiltrating lymphocytes.

### Differential expression of immune checkpoints could alter the prognostic significance of NK and T cells

Chronic activation/inhibition stimuli in the TME can lead to a dysfunctional/exhausted state in tumour-infiltrating NK and T cells, characterized by the expression of various immune checkpoint receptors (65, 66). Hence, we examined the potential association of immune checkpoint receptors with tumour infiltrating NK and T cell signatures in the BLCA TME.

**Figure 6:**
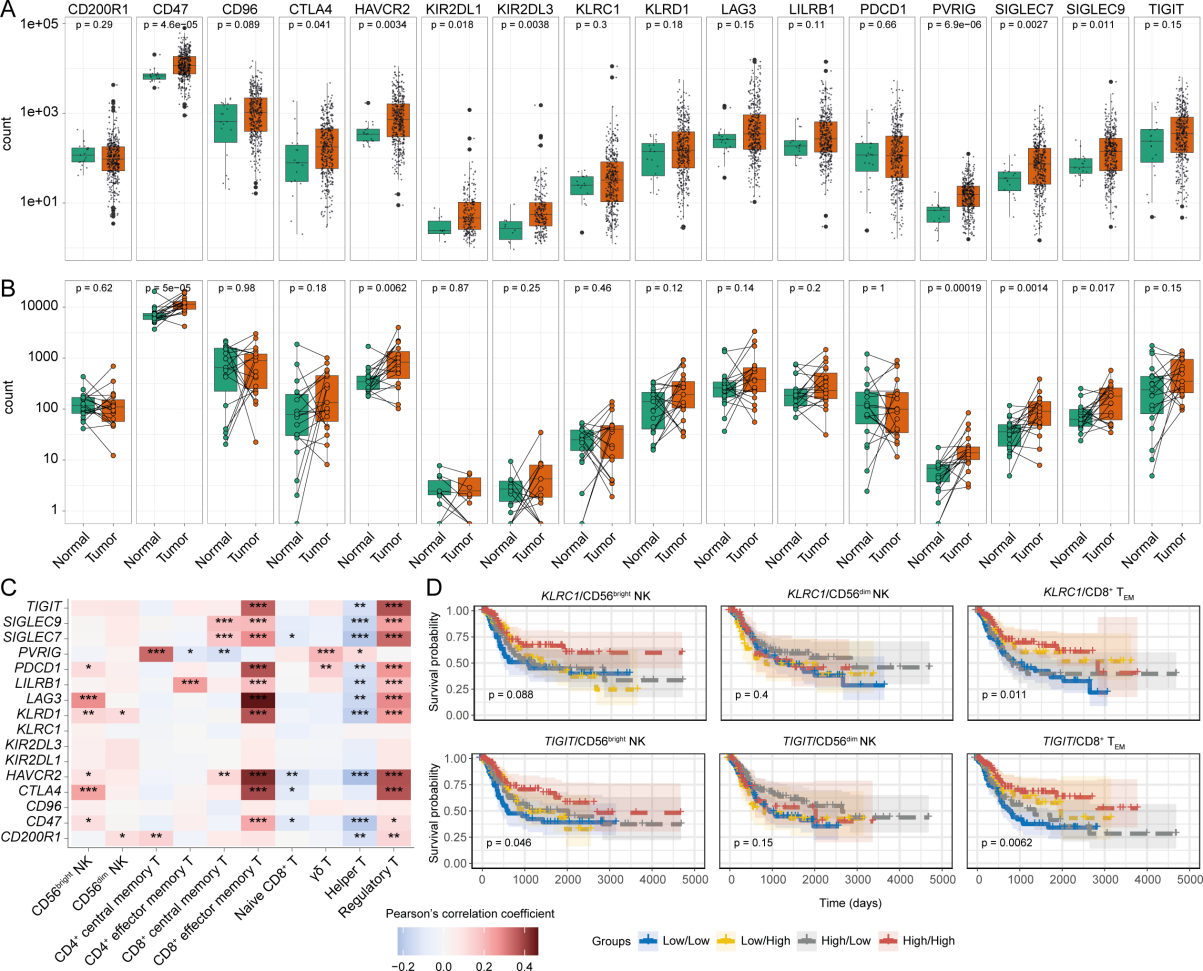
Differentially modulated immune checkpoint receptors can influence the BLCA patient survival. Expression comparison of different immune checkpoints between tumour and tumour-adjacent normal tissues from **(A)** all tumour and normal samples, and **(B)** tumour-matched normal samples; **(C)** Correlation between the expression of the immune checkpoint receptors and abundances of infiltrating NK-T cell subsets, **(D)** KM curves of the immune checkpoint receptor genes KLRC1 (NKG2A), and TIGIT in combination with the NK subsets and CD8^+^ T_EM_ cells. (***p-value < 0.001, **p-value < 0.01, *p-value < 0.05– for Pearson’s correlation coefficient scores).

Firstly, we compared the expression of several immune checkpoint receptor genes between the tumour and adjacent normal tissues. While comparing between matched and unmatched tumour and normal tissue samples, upregulation of checkpoint receptor genes, notably *CD47*, *CTLA4*, *HAVCR2*, *PVRIG*, *SIGLEC7*, and *SIGLEC9*, were specifically detected in malignant compared to normal tissues (**Figures 6A, B**). Furthermore, some of these checkpoint receptors were also positively correlated with tumour infiltrating CD56^bright^ NK and CD8^+^ T_EM_ cell signatures; intriguingly, a similar association with T_reg_ cells and checkpoint receptors was also observed (**Figure 6C**). We next explored the association between immune checkpoint receptor gene expression in patient survival in combination with tumour-infiltrating NK subsets. Combining the NK subset abundances with the expression of checkpoint receptor genes showed little or no significant impact in BLCA patient survival (**Figure 6D**, **Supplementary Figure S10A**). Conversely, there were significant associations with the expression of several checkpoint receptors in combination with the CD8^+^ T_EM_ cell signature and survival, with a marked inverse relationship between higher expression of *KLRC1* (NKG2A), *TIGIT*, *SIGLEC7*, *SIGLEC9*, and *HAVCR2* checkpoint receptors and the CD8^+^ T_EM_ signature on BLCA patient survival (**Figure 6D**, **Supplementary Figure S10A**). Also, most immune checkpoint receptor genes were expressed by cells in the NK/CD8^+^ T cluster of the BLCA scRNA-seq dataset (**Supplementary Figure S10B**). Collectively, these results suggest that expression of immune checkpoint receptors may negatively impact the prognostic implications of CD8^+^ T cells but not NK subsets in BLCA patients.

## Discussion

Natural killer cells were first characterised based on their ability to spontaneously lyse tumour cell-lines, but the clinical significance of a roles for the different NK cell subsets in anti-tumour immunity remains unclear. In this study, we hypothesized that NK cells may exert unique subset-specific responses in the TME which may influence the patient survival outcomes. Through a computational approach, we constructed transcriptional signatures for the functionally distinct CD56^bright^ and CD56^dim^ NK cells and assessed their potential involvement in BLCA patient prognosis using the TCGA cohort. This revealed a subset-specific association of NK cells with tumour surveillance whereby only the CD56^bright^ NK cell signature was correlated with favourable BLCA patient prognosis.

In the linear model of NK cell differentiation, CD56^bright^ NK cells are considered less mature and weakly cytotoxic but potent cytokine-producers with the potential to further differentiate into the more mature and cytotoxic CD56^dim^ NK subset (8, 67). However, how this functionally characteristic dichotomy is maintained during tumour immune surveillance is poorly documented. It has been proposed that complex signaling in the TME may facilitate switching between NK subsets due to the alteration of surface receptor expression patterns (23, 68). We observed a higher abundance of CD56^bright^ than the CD56^dim^ NK subset signatures regardless of clinicopathological stages, with the trend towards progressive loss of CD56^dim^ NK infiltration as the tumour develops. This supports the speculation that prolonged stresses in the TME could lead to a more dysfunctional NK phenotype (69). A similar effect may also occur for the infiltrating CD56^bright^ NK cells, as our data suggests that patients with advanced tumour stages had poor survival despite having a higher NK abundance, suggesting more aggressive forms of BLCA may develop immune escape mechanisms from the infiltrating NK subsets.

NK cell functional responses are determined via signaling through a variety of activating and inhibitory receptors. Thus, we speculated that the expression of NK cell-associated receptors may have clinical relevance for BLCA patients. We detected multiple NK cell family receptor encoding genes (e.g., *KLRK1*, *NCR2*, *CD2*, and *CD160*) which might be critical in mediating anti-tumour immune responses against BLCA because higher expression of these genes was associated with better patient survival probabilities. Furthermore, the expression of these NK cell family receptor genes with the signatures of CD56^bright^ and CD8^+^ T_EM_ cells highlight the potential significance of these receptors in modulating the anti-tumour functionalities of the cells that express them. Further research into the expression and spatial analysis of KLRK1, NCR2, CD2, and CD160 and the cells that express them using multiplex immunohistochemistry may reveal the importance of these receptor-ligand interactions for anti-tumour immunity in the BLCA TME and their association with improved prognosis of BLCA patients.

Within the TME, NK cells are hypothesized to function in a coordinated fashion with other immune cells, particularly DCs, and cytotoxic T lymphocytes (70). Our results provide evidence for a synchronized immune response involving mDCs, CD8^+^ T_EM_ and CD56^bright^ NK cells, which aligns with a putative role for NK-DC crosstalk that may enhance T-cell anti-tumour responses (71, 72). The presence of mDCs, CD8^+^ T_EM_ and CD56^bright^ NK cells were positively correlated with each other, and their combined presence was associated with a favourable BLCA patient survival, which may indicate that the latter cell types may modulate the anti-tumour functionalities of each other. A similar functional NK-DC-CD8^+^ T interaction was observed in pancreatic cancer promoting better patient survival; also, a positive correlation between CD56^high^ NK signature and tissue-residency NK signature was reported (20). In contrast, fibroblasts may negatively impact immune cells by fostering an immunosuppressive tumour microenvironment associated with poor prognosis (73).

While comparing patients with high NK cell abundance against the patients with low NK cell infiltration, we recorded increased expression of many activating and inhibitory NK receptors in the patients with high NK presence. This could indicate the potential presence of NK cells with different functional status (e.g., resting, activated and cytotoxic, and dysfunctional/exhausted NK cells). We speculated that a population of exhausted NK cells might be present in the BLCA TME which may express different immune checkpoint receptors. Early expression of PD-1 (*PDCD1*) marks the activated state on CD8^+^ T cells, whereas late PD-1 expression is more associated with an exhausted T-cell phenotype. Conversely, upregulated CTLA4 expression is only detected in the later stages of CD8^+^ T cell function (74). Our data indicate that patients with higher PD-1 expression and CD8^+^ T_EM_ abundance had better survival potential compared to those with higher PD-1 expression but lower CD8^+^ T_EM_ infiltration. Similar trends were observed for other checkpoint receptors such as LAG3, CD47, CD200R1. Therefore, we speculate that blocking the signaling through these checkpoint receptors at a late stage of BLCA tumour progression could be more effective. On the other hand, we found that patients with lower CTLA4 expression but higher CD8^+^ T_EM_ abundance had better prognosis than the patients with higher CTLA4 and high CD8^+^ T_EM_, rationalizing the CTLA4 blockade at the earlier stage of BLCA, We observed that the patients with higher expression of NKG2A (*KLRC1*) and TIGIT with higher CD56^bright^ NK presence were predicted to have favourable prognosis over the patients with higher expression of these receptors but lower CD56^bright^ NK cells. This could potentially mean that suppressing the signaling from these receptors could reinvigorate the functions of this NK subset (75). Overall, our data revealed that these established immune checkpoint receptors could be novel targets for immune checkpoint blockade, particularly for CD8^+^ T cells in BLCA, however, further experimentations are required to ratify these prospects.

Our analysis also revealed a potential implication of transcription factor *ZNF683* (HOBIT) in modulating the function of NK and T cells in the BLCA TME as well as contributing towards the favourable patient survival. HOBIT is widely considered a master transcription factor that can regulate a program of tissue-residency in lymphocytes (58, 59) and our results indicate the potential presence of tissue-resident subpopulation of NK and T cells in the BLCA TME. Tissue-resident NK cells could be important in eliciting ongoing anti-tumour immunity in the BLCA TME and their identification may provide a novel target for ICB (76). Apart from enhancing the functionality of NK and T cells, IRF3 can also modulate the downstream signaling responses of dendritic cells (77–79), which further indicates the presence of the NK-DC-CD8^+^ crosstalk. Also, we observed a significant combined effect of the mDCs, CD8^+^ T_EM_ and CD56^bright^ NK cell infiltrations on the expression of these TFs and tissue-residency markers in BLCA patients; similar impacts were found on the expression of NK-associated marker genes and their ligands and on numerous immune checkpoint receptor expressions (**Supplementary Figure S11**). Additionally, we observed positive correlations between the expression of *ZNF683* (HOBIT) and the immune checkpoint receptors. Also, patients with low expression of immune checkpoint receptors (i.e. *LAG3*, *HAVCR2*, *CTLA4*, *PDCD1*) and higher expression of *ZNF683* (HOBIT) predicted to be having better survival probabilities, indicating a potential implication of the immune checkpoint receptors on the functions of the tissue-resident NK and CD8^+^ T cell subsets (**Supplementary Figure S12**). Previously, a similar exhausted phenotype was reported for the tissue-resident NK cells in non-small cell lung cancer (17). Overall, these results indicate that all these underlying molecular mechanisms might be happening in a coordinated fashion in the tumour-microenvironment. Despite the intriguing findings, the inherent limitations of computational deconvolution might be present in the analyses. Therefore, further investigation that incorporates a validation cohort coupled with experimental techniques (i.e. immune-histochemistry/flow cytometry) (80) would provide valuable confirmation of these findings.

In summary, our study reveals distinct gene expression patterns associated with tumour infiltrating immune cells. Notably, higher levels of CD56^bright^ NK, T_EM_, and mDC in early-stage BLCA are linked to a more favourable prognosis. Furthermore, we pinpoint specific genes within the CD56^bright^ NK subset, some of which overlap with T_EM_, and demonstrate their prognostic significance, suggesting their potential as markers for monitoring BLCA progression. Targeting these infiltrating lymphocytes and their surface immunomodulatory receptors could enhance the development of subset-specific NK cell therapies.

## Data Availability

The original contributions presented in the study are included in the article/**Supplementary Material**. Further inquiries can be directed to the corresponding authors.

## Supplementary material

Supplementary Figure s1 Cellular hierarchy information for generating the transcriptional signature matrix.

Supplementary Figure s2 Comparison between the estimated fractions of NK subsets in the BLCA patients belonging different clinical-pathological sub-groups of **(A)** Tumour grades, **(B)** Pathological T stage, **(C)** Pathological N staging, **(D)** Pathological M staging, and **(E)** Clinical stages of BLCA. (****p-value < 0.0001, ***p-value < 0.001, **p-value < 0.01, *p-value < 0.05 for Wilcoxon signed-ranked test).

Supplementary Figure s3
**(A)** Abundance of NK subset signatures in BLCA tumour adjacent normal tissue samples. Single cell transcriptome reveals the presence of the NK/CD8^+^ T population in the BLCA TME – **(B)** Clustering of the single cells from all 7 donors shows epithelial cells as the major cell population in BLCA TME; **(C)** Clustering of the CD45^+^ cells; **(D)** SingleR annotation scores for each of the clusters; **(E**) Module scores of CD56^bright^ and CD56^dim^ NK signatures; Comparison between the module scores of NK subsets in cluster 6 against the **(F)** average of all immune cells, and **(G)** specific module scores for other immune cells; **(H)** Cell-cell communication networks between the immune cell clusters; Signaling **(I)** towards cluster 6 cells, and (J) from cluster 6 from/to the other immune cell clusters. (****p-value < 0.0001, ***p-value < 0.001, **p-value < 0.01, *p-value < 0.05 for Wilcoxon signed-ranked test).

Supplementary Figure s4 Multivariate Cox regression analysis between the covariates non-silent mutational load, CD56^bright^ NK abundance, and total CD8^+^ T cell abundance in TCGA-BLCA patient transcriptomes.

Supplementary Figure S5 Patients from different clinical stages **(A)** histologic grade, **(B)** Pathologic T staging, **(C)** Pathologic N staging, **D.** Pathologic M staging, **(E)** Pathologic stage show diverse survival responses in accordance with the infiltrating NK subsets.

Supplementary Figure S6
**(A)** Multivariate Cox regression analysis between the covariates CD56^bright^ NK abundance, and abundance of NK cell receptor transcripts in TCGA-BLCA patient transcriptomes. Association of the expression of ligands for the NK-receptors with the infiltrating NK subsets. **(B)** KM curves for *KLRK1* (NKG2D), *NCR2* (NKp44), *CD2*, and *CD160* receptor genes in combination with CD8^+^ T_EM_ cells. **(C)** Correlation heatmaps for showing the correlation between ligands of different NK receptors and NK/T cell subsets; **(D)** KM curves to highlight the prognostic significance of ligand-receptor pairs of *KLRK1* (NKG2D), *NCR2* (NKp44), *CD2*, and *CD160*. **(E)** Expression comparison of the receptor encoding transcripts between tumour and tumour-adjacent normal tissues. **(F)** Expression of the NK-associated markers in the scRNA-seq dataset. (****p-value < 0.0001, ***p-value < 0.001, **p-value < 0.01, *p-value < 0.05– for Wilcoxon signed-ranked test or Pearson’s correlation coefficient scores).

Supplementary Figure S7
**(A)** KNN graph and Louvain community clustering of the BLCA patients based on the 3 features (CD56^bright^ NK, CD8^+^ T_EM_ and mDC fractions obtained from the deconvolution of TCGA-BLCA bulk RNA-seq dataset). Mapping of the single gene set module scores (performed scoring by “singscore” algorithm) of – **(B)** CD56^bright^ NK, **(C)** CD8^+^ T_EM_ and **(D)** mDC cell signatures on to the BLCA patient clusters generated. **(E)** Multiple linear regression model assessing the collinearity of the independent gene set scores from singscore for CD56^bright^ NK, CD8^+^ T_EM_ and mDC cells.

Supplementary Figure S8 Functional pathways associated with the differentially expressed genes of each cluster of CD45^+^ cells in the analysed scRNA-seq dataset.

Supplementary Figure S9
**(A)** Expression comparison of the TF encoding transcripts between tumour and tumour-adjacent normal tissues. **(B)** Prognostic associations of IRF3 in combination with NK subsets and CD8^+^ T_EM_ cell. (**C)** Multivariate Cox regression analysis between the covariates CD56^bright^ NK abundance, and HOBIT (*ZNF683*) encoding transcripts in TCGA-BLCA patient transcriptomes. Functional pathways associated with **(D)** STAT2, and **(E)** IRF3 expression. **(F)** Correlation heatmap between the expression of the TFs with NK associated receptors. **(G)** Expression of the tissue-residency markers in the immune cell clusters of the analysed scRNA-seq dataset.

Supplementary Figure S10
**(A)** KM curves of the immune checkpoint receptor genes in combination with the NK subsets and CD8^+^ T_EM_ cells. **(B)** Expression of the checkpoint receptors in the different clusters of CD45^+^ cells in the BLCA scRNA-seq dataset.

Supplementary Figure S11 Multiple linear regression model assessing the collective impact of infiltration abundances of CD56^bright^ NK, CD8^+^ T_EM_, and mDC on the expression of **(A)** NK-associated receptors, **(B)** ligands of several key NK receptors, **(C)** TFs and tissue-residency factors, and **(D)** Immune checkpoint receptors. Heatmaps represents the p-values from the t-statistics of the individual effect of each of the cell-types abundance on the expression of the genes, the asterisks above each row indicate the P-values from the F-statistics of the linear model for the combined effect of cell-type abundances on gene expression (****p-value < 0.0001, ***p-value < 0.001, **p-value < 0.01, *p-value < 0.05).

Supplementary Figure S12
**(A)** Correlation between the expression of *ZNF683* and other tissue-residency factors with the expression of immune checkpoint receptor encoding genes. **(B)**. Combination KM curves between *ZNF683* (HOBIT) and the immune checkpoint receptors.
