# Supplementary figures and images for: Transcriptional signature of CD56bright NK cells predicts favourable prognosis in bladder cancer
Source: Front Immunol. 2025 Jan 14;15:1474652. doi: 10.3389/fimmu.2024.1474652 (PMC11772185; doi:10.3389/fimmu.2024.1474652)

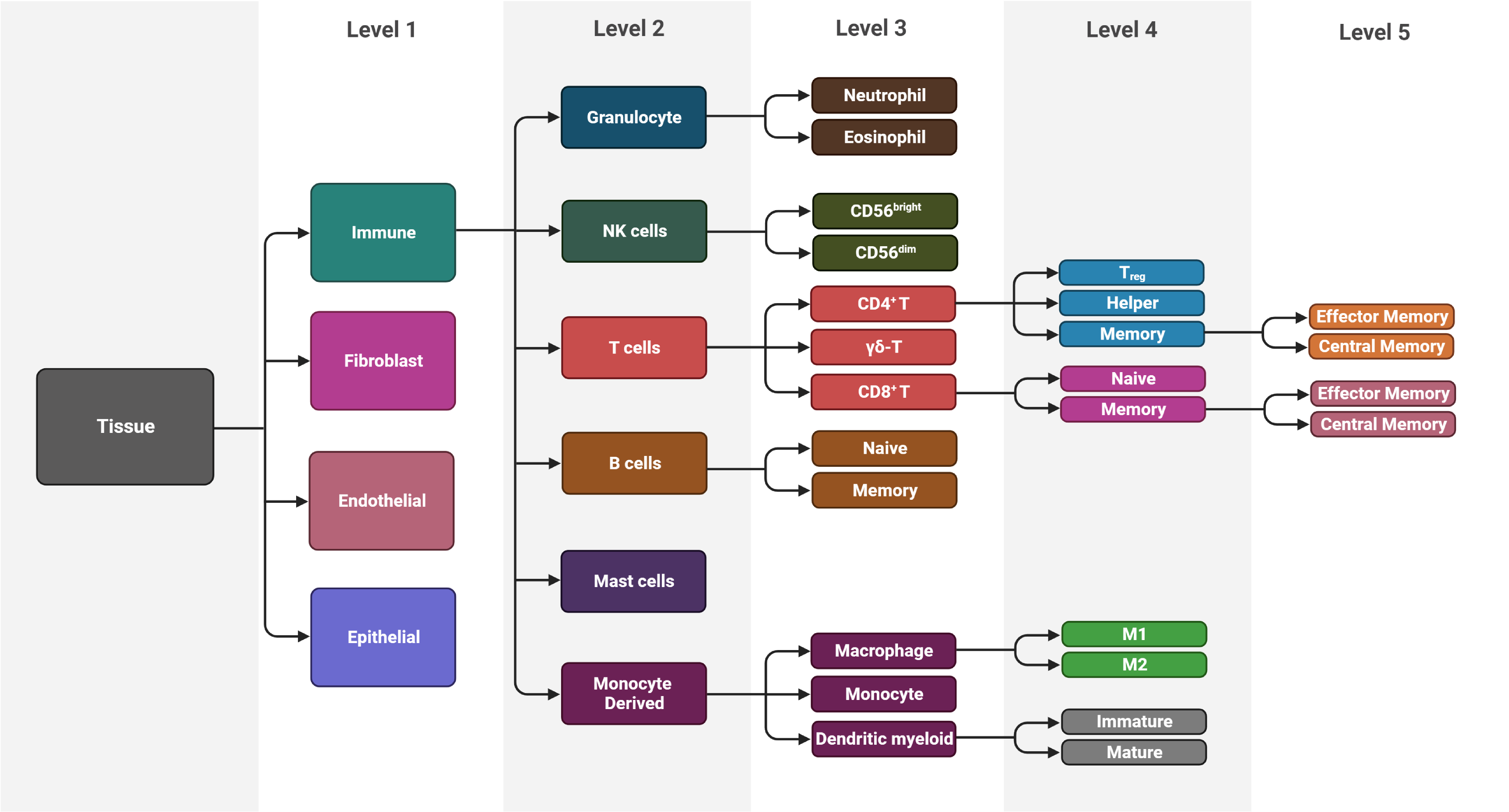

Supplement: Supplementary Figure s1 — Cellular hierarchy information for generating the transcriptional signature matrix. [file Image1.tif]

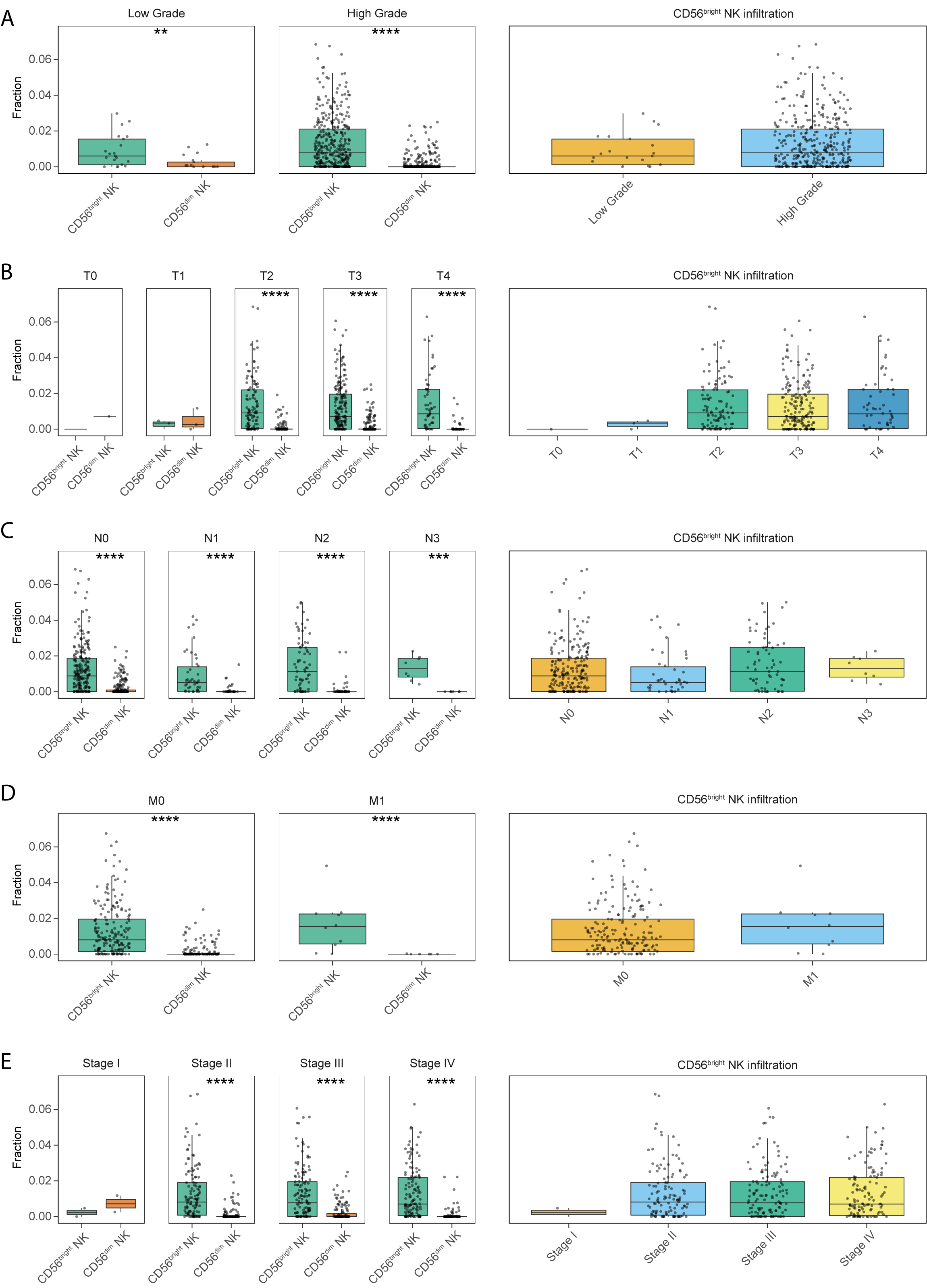

Supplement: Supplementary Figure s2 — Comparison between the estimated fractions of NK subsets in the BLCA patients belonging different clinical-pathological sub-groups of (A) Tumour grades, (B) Pathological T stage, (C) Pathological N staging, (D) Pathological M staging, and (E) Clinical stages of BLCA. (****p-value < 0.0001, ***p-value < 0.001, **p-value < 0.01, *p-value < 0.05 for Wilcoxon signed-ranked test). [file Image2.tif]

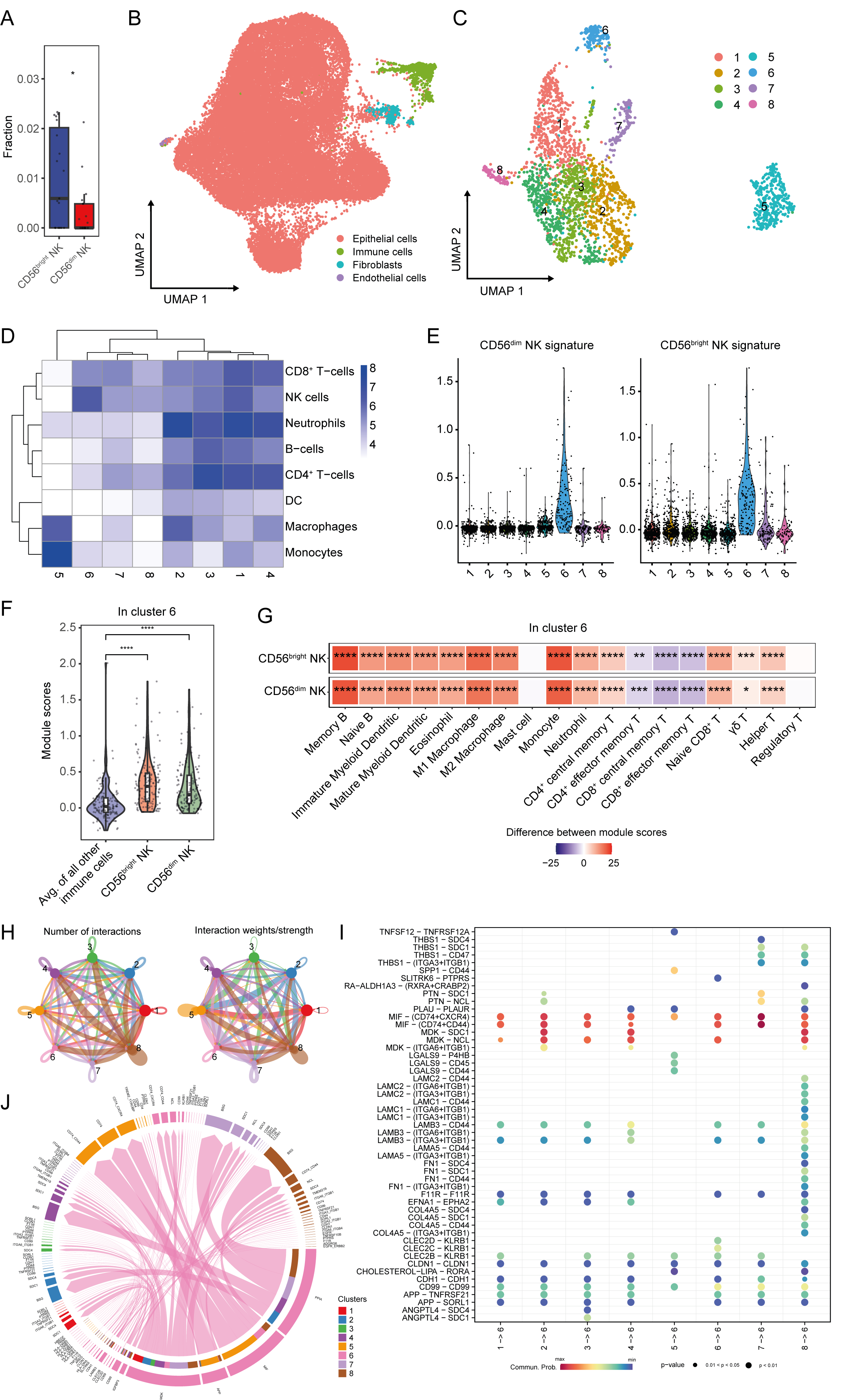

Supplement: Supplementary Figure s3 — (A) Abundance of NK subset signatures in BLCA tumour adjacent normal tissue samples. Single cell transcriptome reveals the presence of the NK/CD8+ T population in the BLCA TME – (B) Clustering of the single cells from all 7 donors shows epithelial cells as the major cell population in BLCA TME; (C) Clustering of the CD45+ cells; (D) SingleR annotation scores for each of the clusters; (E) Module scores of CD56bright and CD56dim NK signatures; Comparison between the module scores of NK subsets in cluster 6 against the (F) average of all immune cells, and (G) specific module scores for other immune cells; (H) Cell-cell communication networks between the immune cell clusters; Signaling (I) towards cluster 6 cells, and (J) from cluster 6 from/to the other immune cell clusters. (****p-value < 0.0001, ***p-value < 0.001, **p-value < 0.01, *p-value < 0.05 for Wilcoxon signed-ranked test). [file Image3.tif]

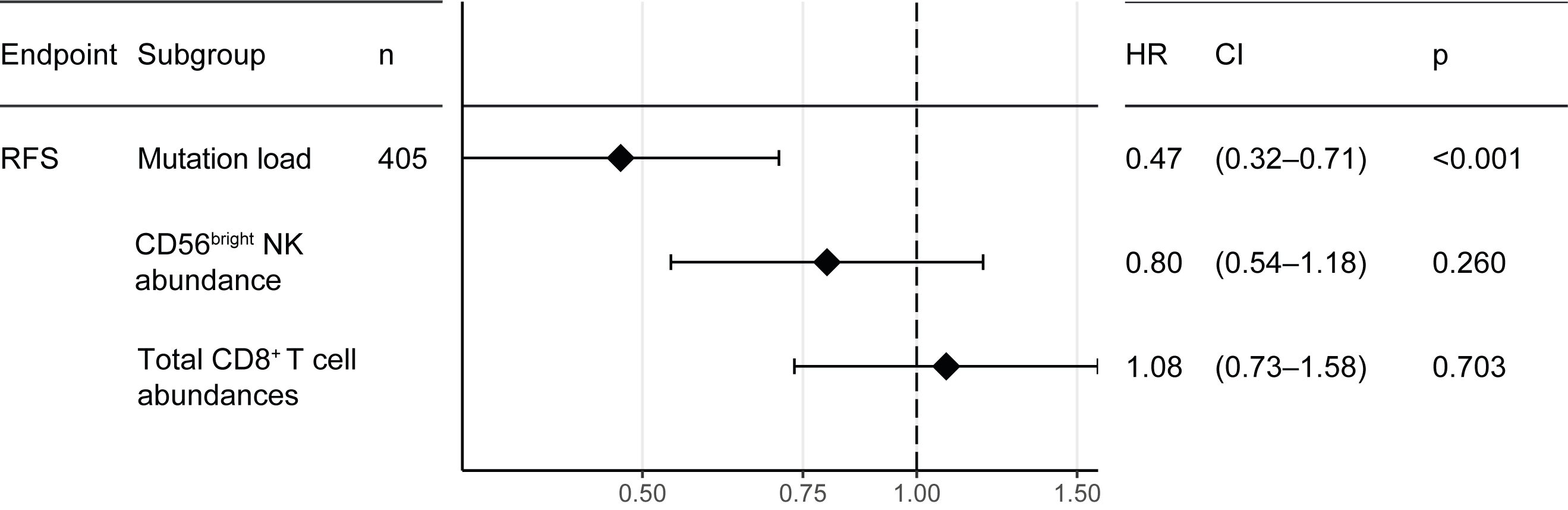

Supplement: Supplementary Figure s4 — Multivariate Cox regression analysis between the covariates non-silent mutational load, CD56bright NK abundance, and total CD8+ T cell abundance in TCGA-BLCA patient transcriptomes. [file Image4.tif]

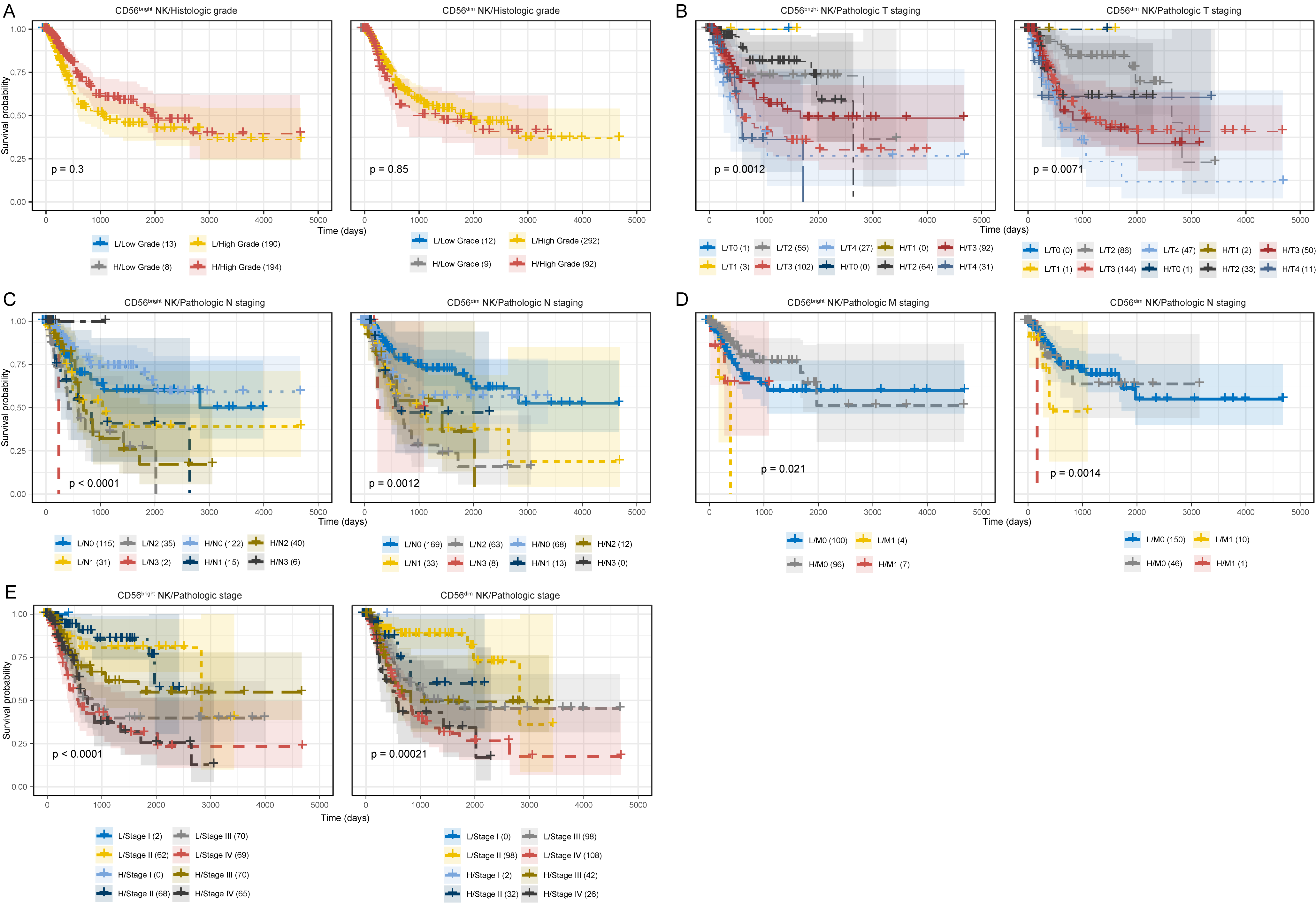

Supplement: Supplementary Figure S5 — Patients from different clinical stages (A) histologic grade, (B) Pathologic T staging, (C) Pathologic N staging, D. Pathologic M staging, (E) Pathologic stage show diverse survival responses in accordance with the infiltrating NK subsets. [file Image5.tif]

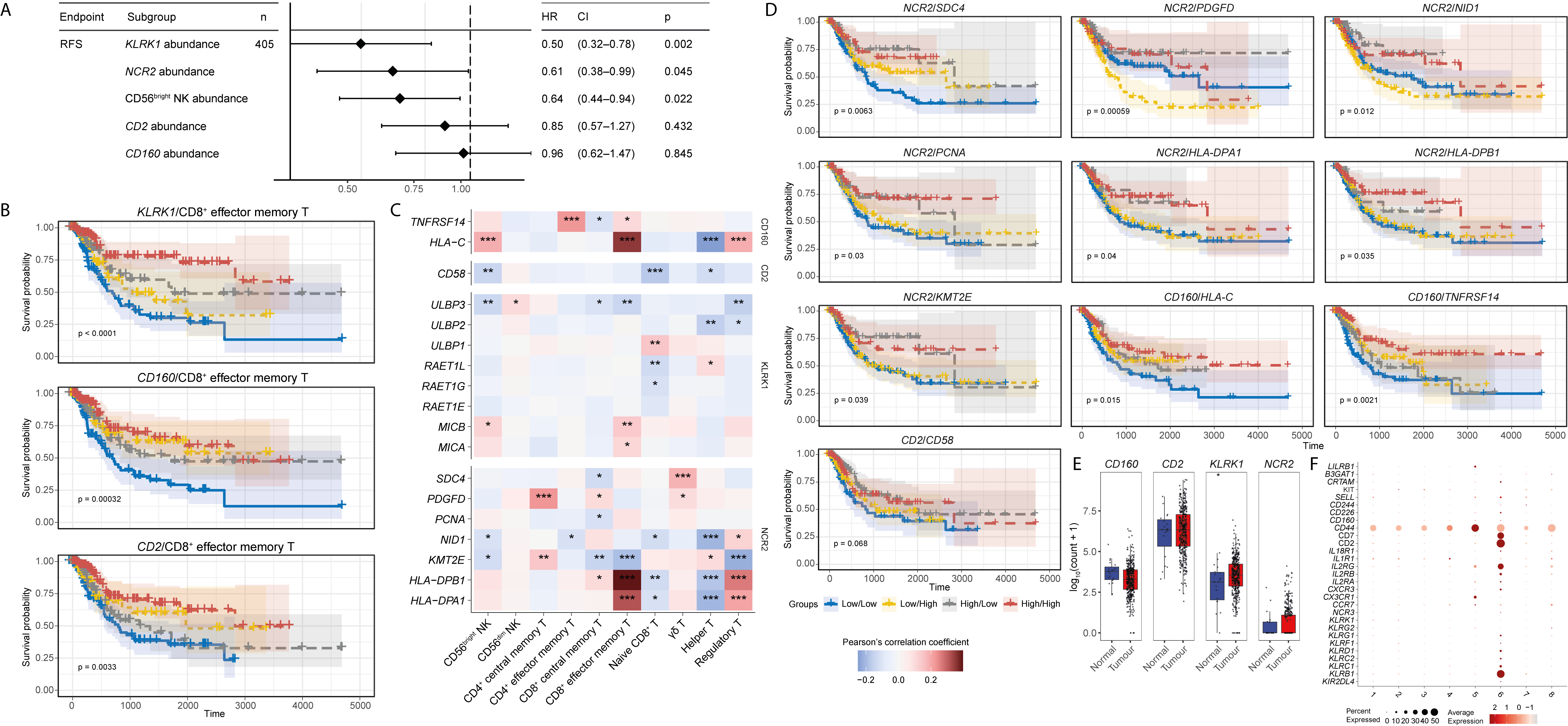

Supplement: Supplementary Figure S6 — (A) Multivariate Cox regression analysis between the covariates CD56bright NK abundance, and abundance of NK cell receptor transcripts in TCGA-BLCA patient transcriptomes. Association of the expression of ligands for the NK-receptors with the infiltrating NK subsets. (B) KM curves for KLRK1 (NKG2D), NCR2 (NKp44), CD2, and CD160 receptor genes in combination with CD8+ TEM cells. (C) Correlation heatmaps for showing the correlation between ligands of different NK receptors and NK/T cell subsets; (D) KM curves to highlight the prognostic significance of ligand-receptor pairs of KLRK1 (NKG2D), NCR2 (NKp44), CD2, and CD160. (E) Expression comparison of the receptor encoding transcripts between tumour and tumour-adjacent normal tissues. (F) Expression of the NK-associated markers in the scRNA-seq dataset. (****p-value < 0.0001, ***p-value < 0.001, **p-value < 0.01, *p-value < 0.05– for Wilcoxon signed-ranked test or Pearson’s correlation coefficient scores). [file Image6.tif]

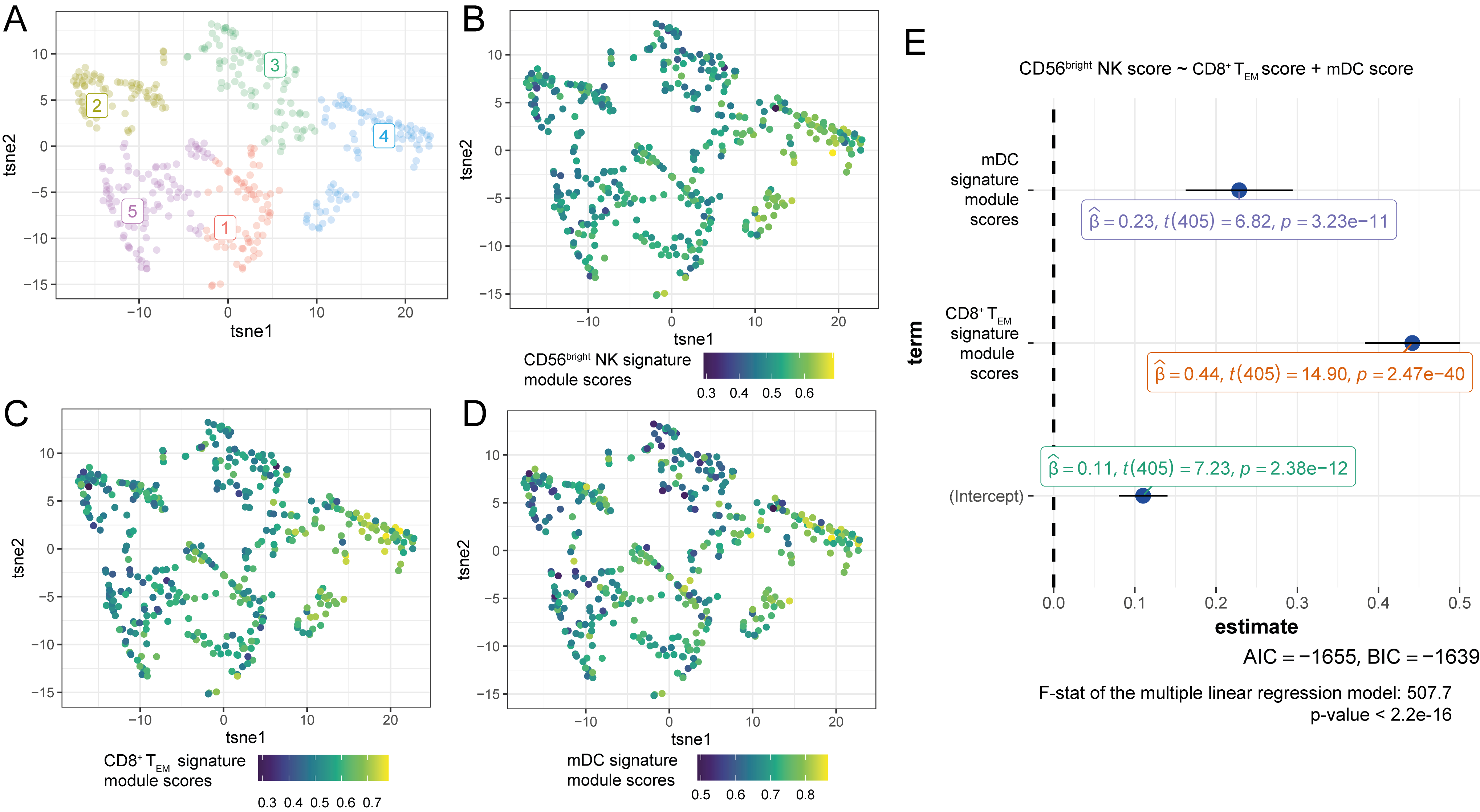

Supplement: Supplementary Figure S7 — (A) KNN graph and Louvain community clustering of the BLCA patients based on the 3 features (CD56bright NK, CD8+ TEM and mDC fractions obtained from the deconvolution of TCGA-BLCA bulk RNA-seq dataset). Mapping of the single gene set module scores (performed scoring by “singscore” algorithm) of – (B) CD56bright NK, (C) CD8+ TEM and (D) mDC cell signatures on to the BLCA patient clusters generated. (E) Multiple linear regression model assessing the collinearity of the independent gene set scores from singscore for CD56bright NK, CD8+ TEM and mDC cells. [file Image7.tif]

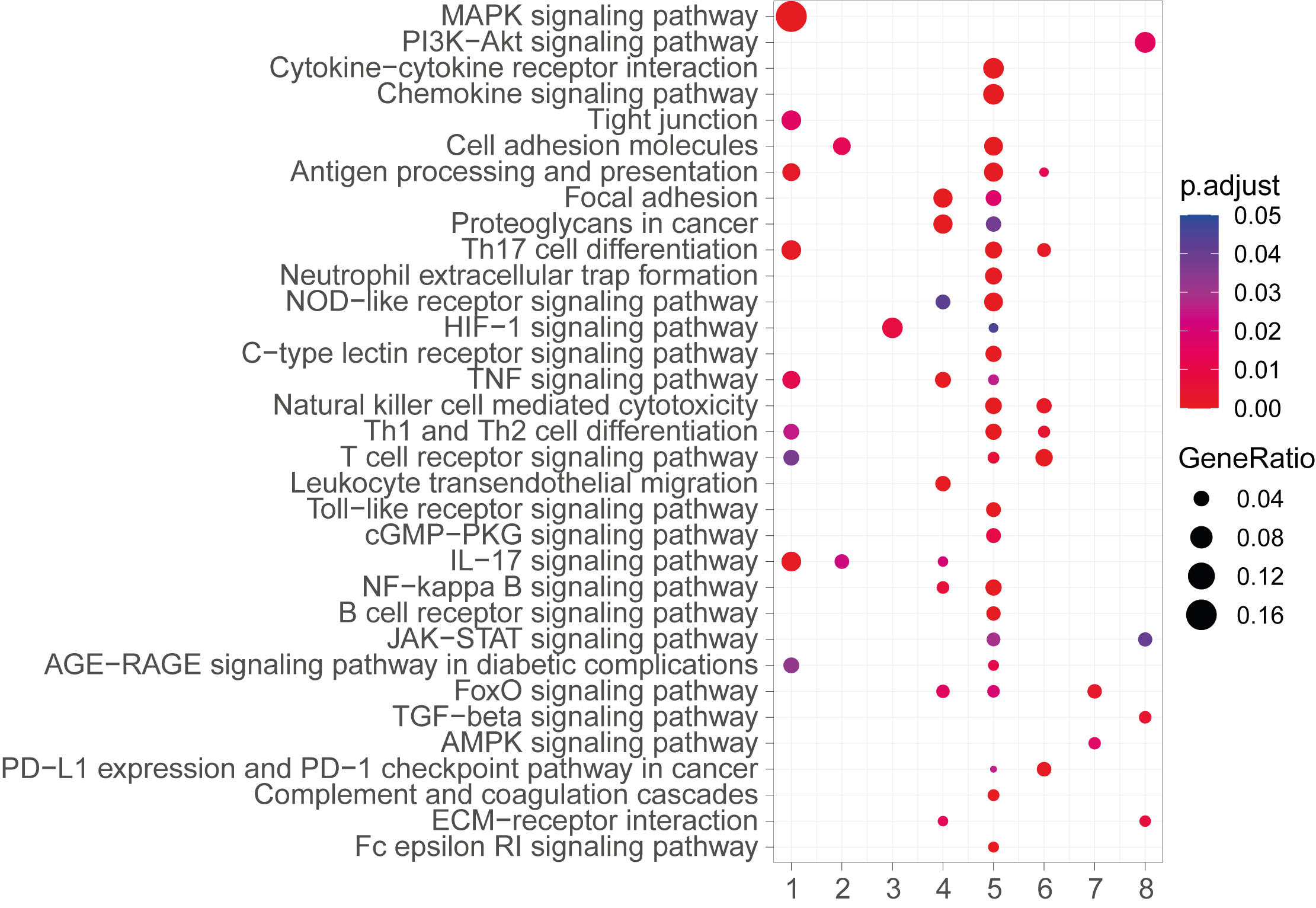

Supplement: Supplementary Figure S8 — Functional pathways associated with the differentially expressed genes of each cluster of CD45+ cells in the analysed scRNA-seq dataset. [file Image8.tif]

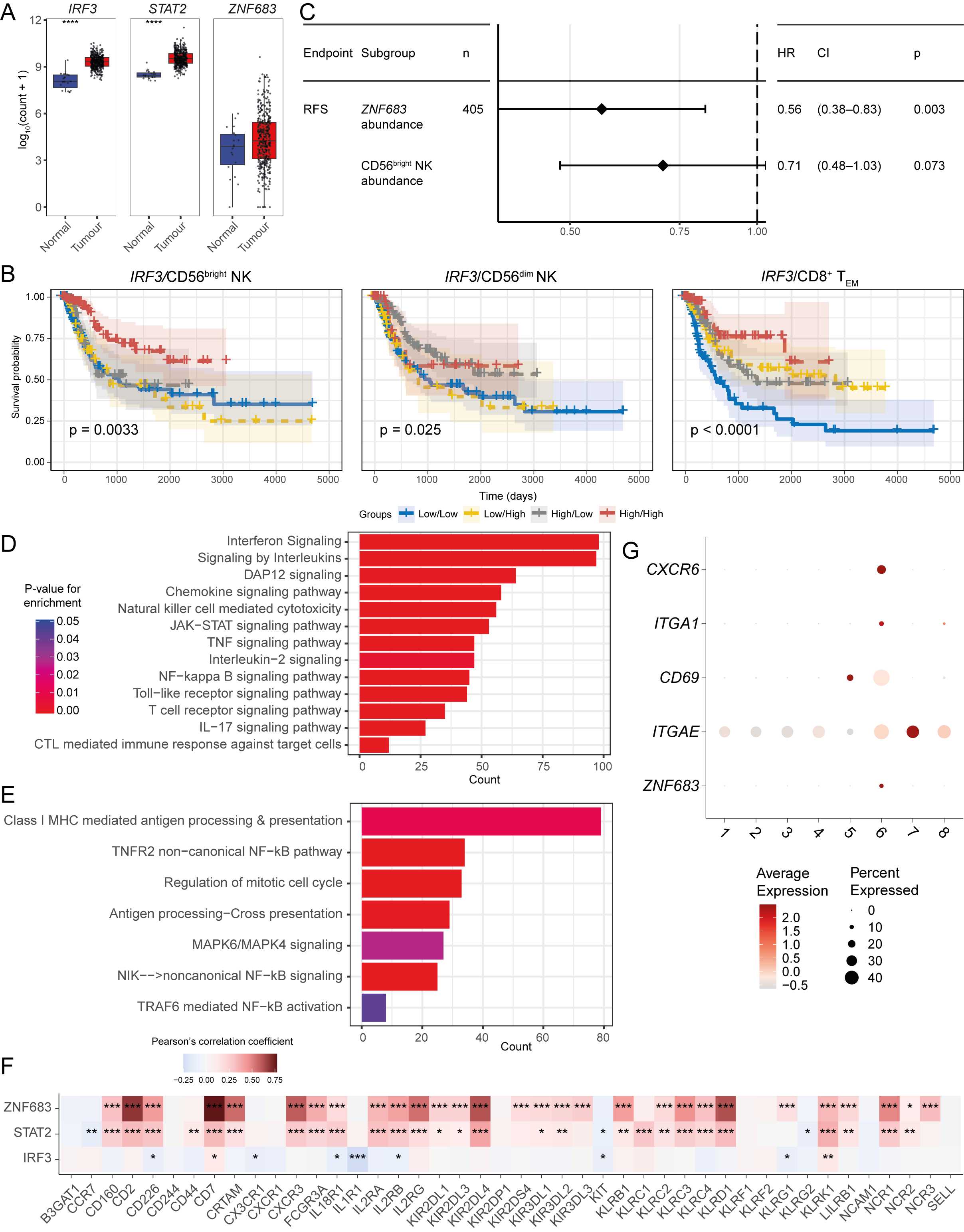

Supplement: Supplementary Figure S9 — (A) Expression comparison of the TF encoding transcripts between tumour and tumour-adjacent normal tissues. (B) Prognostic associations of IRF3 in combination with NK subsets and CD8+ TEM cell. (C) Multivariate Cox regression analysis between the covariates CD56bright NK abundance, and HOBIT (ZNF683) encoding transcripts in TCGA-BLCA patient transcriptomes. Functional pathways associated with (D) STAT2, and (E) IRF3 expression. (F) Correlation heatmap between the expression of the TFs with NK associated receptors. (G) Expression of the tissue-residency markers in the immune cell clusters of the analysed scRNA-seq dataset. [file Image9.tif]

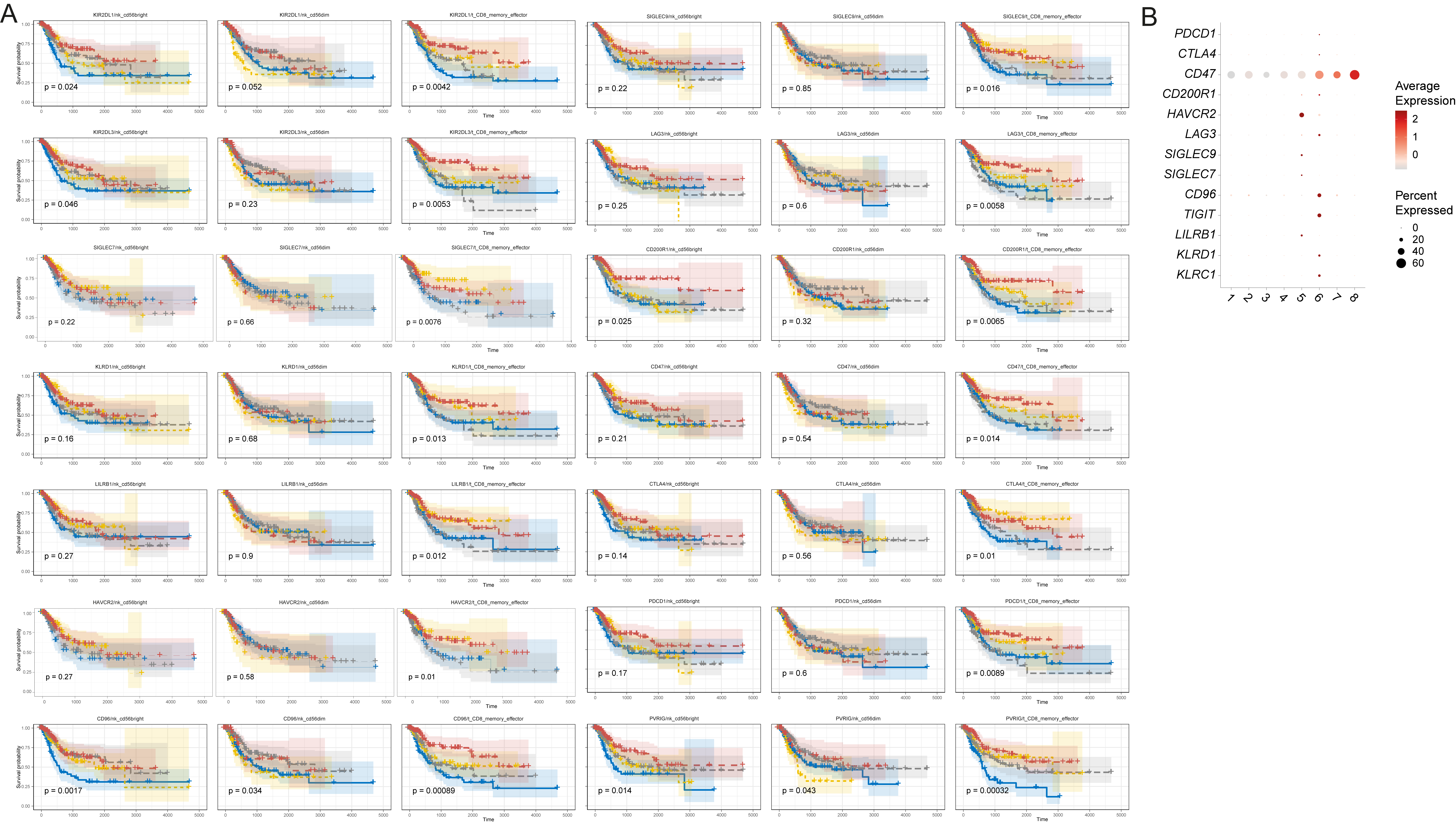

Supplement: Supplementary Figure S10 — (A) KM curves of the immune checkpoint receptor genes in combination with the NK subsets and CD8+ TEM cells. (B) Expression of the checkpoint receptors in the different clusters of CD45+ cells in the BLCA scRNA-seq dataset. [file Image10.tif]

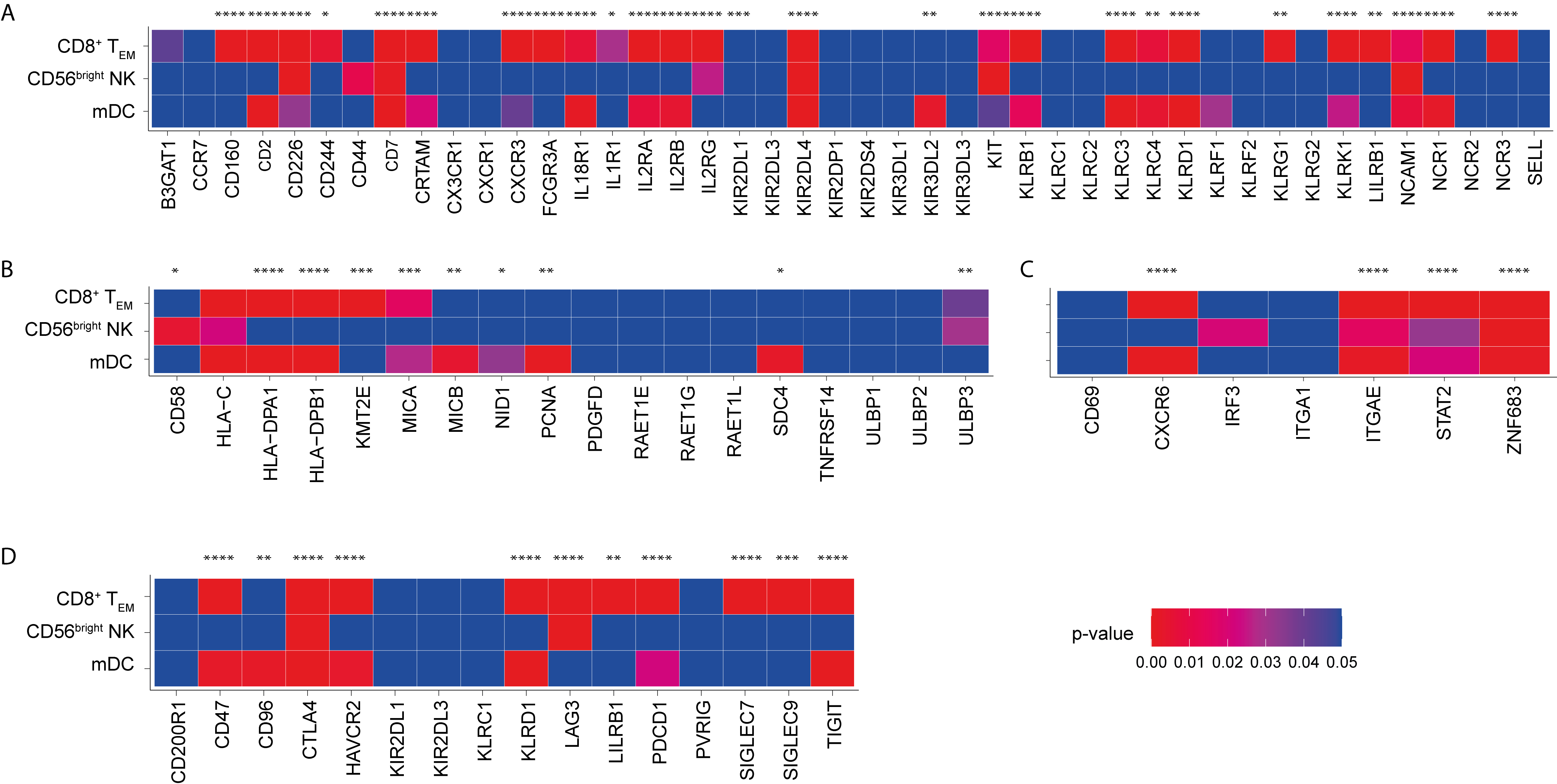

Supplement: Supplementary Figure S11 — Multiple linear regression model assessing the collective impact of infiltration abundances of CD56bright NK, CD8+ TEM, and mDC on the expression of (A) NK-associated receptors, (B) ligands of several key NK receptors, (C) TFs and tissue-residency factors, and (D) Immune checkpoint receptors. Heatmaps represents the p-values from the t-statistics of the individual effect of each of the cell-types abundance on the expression of the genes, the asterisks above each row indicate the P-values from the F-statistics of the linear model for the combined effect of cell-type abundances on gene expression (****p-value < 0.0001, ***p-value < 0.001, **p-value < 0.01, *p-value < 0.05). [file Image11.tif]

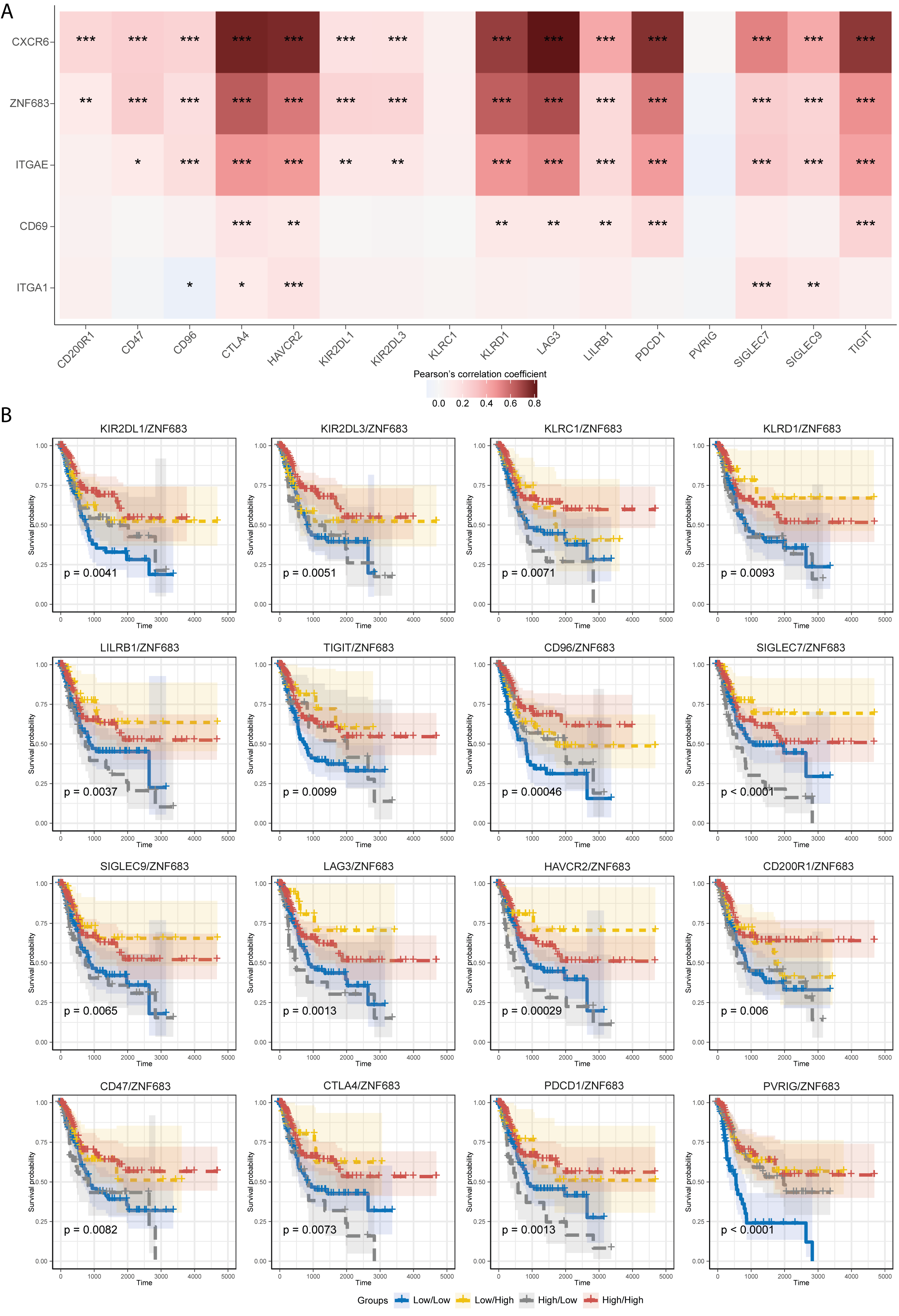

Supplement: Supplementary Figure S12 — (A) Correlation between the expression of ZNF683 and other tissue-residency factors with the expression of immune checkpoint receptor encoding genes. (B). Combination KM curves between ZNF683 (HOBIT) and the immune checkpoint receptors. [file Image12.tif]
